# HSP90A inhibition promotes anti-tumor immunity by reversing multi-modal resistance and stem-like property of immune-refractory tumors

**DOI:** 10.1038/s41467-019-14259-y

**Published:** 2020-01-28

**Authors:** Kwon-Ho Song, Se Jin Oh, Suyeon Kim, Hanbyoul Cho, Hyo-Jung Lee, Joon Seon Song, Joon-Yong Chung, Eunho Cho, Jaeyoon Lee, Seunghyun Jeon, Cassian Yee, Kyung-Mi Lee, Stephen M. Hewitt, Jae-Hoon Kim, Seon Rang Woo, Tae Woo Kim

**Affiliations:** 1 Department of Biochemistry & Molecular Biology, Korea University College of Medicine, Seoul, Korea; 2 Department of Biomedical Science, College of Medicine, Korea University College of Medicine, Seoul, Korea; 3 Translational Research Institute for Incurable Diseases, Korea University College of Medicine, Seoul, Korea; 4 Department of Obstetrics and Gynecology, Gangnam Severance Hospital, Yonsei University College of Medicine, Seoul, Korea; 5 Experimental Pathology Laboratory, Laboratory of Pathology, Center for Cancer Research, National Cancer Institute, National Institutes of Health, Bethesda, MD 20892 USA; 6 Department of Pathology, Asan Medical Center, University of Ulsan College of Medicine, Seoul, 06351 Korea; 7 College of Science, College of Social Sciences and Humanities, Northeastern University, Boston, MA USA; 8 Department of Gynecologic Oncology, The University of Texas MD Anderson Cancer Center, Houston, TX USA

**Keywords:** Cancer, Cancer stem cells, Cancer therapy, Tumour immunology

## Abstract

Cancer immunotherapy has emerged as a promising cancer treatment. However, the presence of immune-refractory tumor cells limits its clinical success by blocking amplification of anti-tumor immunity. Previously, we found that immune selection by immunotherapy drives the evolution of tumors toward multi-modal resistant and stem-like phenotypes via transcription induction of AKT co-activator TCL1A by NANOG. Here, we report a crucial role of HSP90A at the crossroads between NANOG-TCL1A axis and multi-aggressive properties of immune-edited tumor cells by identifying HSP90AA1 as a NANOG transcriptional target. Furthermore, we demonstrate that HSP90A potentiates AKT activation through TCL1A-stabilization, thereby contributing to the multi-aggressive properties in NANOG^high^ tumor cells. Importantly, HSP90 inhibition sensitized immune-refractory tumor to adoptive T cell transfer as well as PD-1 blockade, and re-invigorated the immune cycle of tumor-reactive T cells. Our findings implicate that the HSP90A-TCL1A-AKT pathway ignited by NANOG is a central molecular axis and a potential target for immune-refractory tumor.

## Abstract (web summary)

Nanog can confer resistance to cancer immunotherapy by promoting AKT activity. Here, the authors demonstrate that HSP90A is a Nanog target that stabilizes the AKT coactivator TCL1, thereby activating AKT, and that HSP90A inhibition can enhance the anti-tumor efficacy of adoptive T cell transfer and checkpoint blockade.

## Introduction

Harnessing the immune system to detect and eliminate tumor cells, cancer immunotherapy has emerged as a potentially powerful approach to cancer treatment^1^. Particularly, T cell-based therapeutic methods such as adoptive T cell transfer (ACT) and immune checkpoint blockades (ICB) have achieved tremendous progress in the field of cancer immunotherapy. However, despite the developing field’s potential to revolutionize cancer treatment, the presence of immunotherapeutic resistance limits its clinical application^2^. Among the diverse causes of resistance to immunotherapy, the cancer immunoediting theory has attracted attention as it can explain the emergence of resistance to anti-tumor immunity^3^. Indeed, there is increasing evidence that cancer immunoediting drives the adaptation of tumor cells to host immune surveillance, thereby contributing to a generation of cancer cells with better survival advantages^4^. Furthermore, previous studies provide evidence that preferential selection and subsequent expansion of a subset of stem-like tumor cells with an undifferentiated phenotype contribute to therapeutic resistance^5–7^. In this regard, we had found that immune selection by immunotherapy drives the malignant evolution of tumors toward multi-modal resistance and stem-like phenotypes^8^. However, the potential link between the host immune-intrinsic mechanisms (i.e., cancer immunoediting) and the development of the malignant phenotypes of immune-refractory tumor cells is not well understood.

Accumulating evidence indicate that the intrinsic properties of tumors not only promote tumorigenesis but also interfere with processes essential for an effective anti-tumor immune response, such as T cell trafficking to tumors and T cell-mediated killing of tumor cells^9–11^. It has been documented that tumor cell death by T cells could lead to release of tumor antigens that prime subsequent immune responses, known as the cancer-immunity cycle^12^. Furthermore, recent studies provide evidence that T cell-mediated killing of tumor cells is important for initiating or re-invigorating the cancer-immunity cycle providing stimuli of tumor neo-antigens to cytotoxic T lymphocytes (CTLs)^13,14^, suggesting that intrinsic resistance of tumor cells to CTLs is a critical obstacle to improving cancer immunotherapy. In this regard, we have identified the transcription factor (TF) NANOG as a key intrinsic factor that renders immune-edited tumor cells impervious to cytotoxicity of T cells^15,16^. Therefore, NANOG, an intrinsic factor of tumor cells, is a potential target to overcome the immune-refractoriness by re-invigorating the immune cycle of tumor-reactive T cells. In addition, we have also found that NANOG confers multi-aggressive phenotypes to tumor cells through transcriptional induction of TCL1A and subsequent activation of the AKT-signaling pathway^16^. Notably, the inhibition of the NANOG signaling caused reversal of immune-resistant phenotypes of the tumor cells and led to long-term control of the disease^16^. This suggest that the strategies impeding the NANOG–TCL1A–AKT axis may not only conquer the problem of immune escape but also that of multi-aggressive properties in immune-refractory cancer. Given that clinically available pharmacologic inhibitors for NANOG have not been developed yet, the identification of an additional targetable pathway is needed to potentiate the efficacy of immunotherapy.

Heat shock protein 90 (HSP90), a molecular chaperone which assists in protein folding to reach stability or degradation, is known for its up-regulation in a wide range of cancers and close association with a poor prognosis and resistance to chemotherapies and radio-therapies^17,18^. In mammalian cells, HSP90 consists of four major subtypes: the inducible cytosolic isoforms of HSP90A encoded by HSP90AA1, the constitutively expressed HSP90B encoded by HSP90AB1, endoplasmic reticulum GRP94 encoded by HSP90B1, and mitochondrial TRAP-1 encoded by TRAP1 (ref. ^19^). Of the HSP90s, HSP90A is overexpressed and increases multi-malignant phenotypes including chemo-resistance to cisplatin as well as metastatic potentials in various types of cancers^19–21^. Although the importance of HSP90A as a therapeutic target continues to grow, the potential relationship between HSP90A and the multi-aggressive phenotypes conferred by the NANOG–TL1A–AKT axis is yet to be studied extensively.

Here, we report that HSP90A is a clinically actionable target for NANOG-mediated multi-aggressive properties of immune-edited tumor cells. Mechanistically, transcriptional induction of HSP90AA1 by NANOG leads to stabilization of TCL1A, which contributes to subsequent activation of the AKT-signaling pathway. Furthermore, we demonstrate that HSP90A inhibition with AUY-922 renders tumor susceptible to T cell-based immunotherapy including ACT and anti-PD-1 therapy, and leads to increase of the infiltration of tumor-reactive T cells via amplification of anti-tumor immunity. Thus, we provide proof of principle in a preclinical model that the inhibition of HSP90A signaling is an appealing therapeutic strategy to incorporate with various cancer therapeutic modalities, particularly an immune-based modality, and overcome NANOG^high^ immune-refractory tumors.

## Results

### HSP90A confers multi-modal resistance and stem-like property

**Fig. 1:**
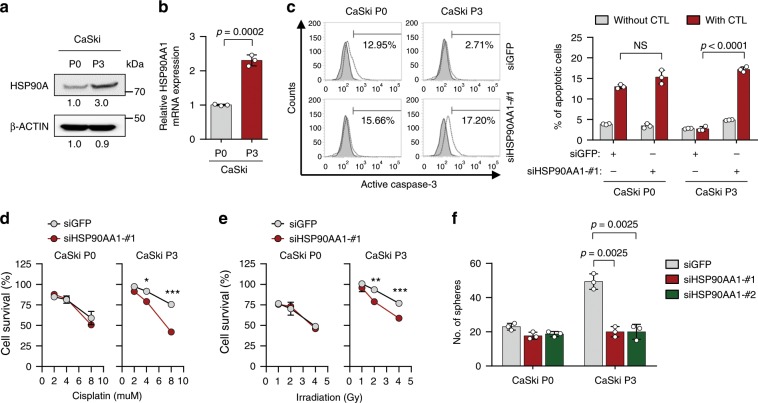
HSP90A is required for multi-aggressive properties of immune-edited tumor cells. **a** HSP90A protein level in CaSki P0 and P3 cells was determined by Western blot. β-ACTIN was included as an internal loading control. Numbers below blot images indicate the expression as measured by fold change. **b** HSP90AA1 mRNA level in CaSki P0 and P3 cells was determined by qRT-PCR. **c–e** CaSki P0 and P3 cells were transfected with siGFP or siHSP90AA1-#1. **c** The frequency of apoptotic (active caspase-3^+^) cells in the MART-1 peptide pulsed cells after incubation with or without MART-1-specific CTLs at a 1:1 ratio for 4 h was estimated by flow cytometry analysis. **d** and **e** Cells were treated with indicated concentrations of cisplatin **d** and irradiation **e**. The percentage of viable cells was determined by trypan blue exclusion assay at 24 h after either cisplatin treatment or irradiation. *p < 0.01, **p < 0.001, and ***p < 0.0001. **f** CaSki P0 and P3 cells were transfected with siGFP, siHSP90AA1-#1, or siHSP90AA1-#2. Sphere-forming capacity of the cells in low-density suspension culture. All experiments were performed in triplicate. The *p*-values by two-tailed Student’s *t* test **b** or two-way ANOVA **c–f** are indicated. NS, not significant. Data represent the mean ± SD. Source data are provided as a Source Data file.

Previously, we established a highly immune-resistant cervical tumor cell line, CaSki P3, generated from its immune susceptible parental cell line, CaSki P0, through three rounds of selection by cognate CTLs^22^. Notably, the immune-edited tumor cells (termed P3) were refractory to apoptotic death by multi-modality including cisplatin, γ-radiation, as well as cognate CTLs, whereas the parental cells (termed P0) remained sensitive to these^8^. To investigate potential targetable pathways for restoring sensitivity to multi-modalities in the immune-edited tumor cells, we performed 2D protein electrophoresis and mass spectrometry in lysates derived from P0 and P3 cells. From this analysis, we noted that HSP90A was up-regulated in P3 cells compared to P0 cells (Supplementary Fig. 1); this was then confirmed by Western blot (Fig. 1a). The increased level of HSP90A protein in P3 cells was accompanied by HSP90AA1 mRNA expression (Fig. 1b). HSP90AB1 and HSP90B1 encoding HSP90B and GRP-94, respectively, showed no significant changes in mRNA expression from P0 and P3 cells (Supplementary Fig. 2), further specifying HSP90A as our subjectable molecule. To determine the roles of HSP90AA1 up-regulation in the immune-resistant phenotype of P3 tumor cells, we silenced HSP90AA1 in P3 cells using three kinds of siRNAs: siHSP90AA1 #1, #2, or #3 (Supplementary Fig. 3a). Compared to siGFP-transfected control cells, siHSP90AA1s-transfected P3 cells are more susceptible to apoptosis induced by granzyme B, a key component in CTL-mediated apoptosis (Supplementary Fig. 3b). Consistently, silencing of HSP90AA1 reverses resistant phenotypes against to cognate CTLs of P3 tumor cells (Fig. 1c), indicating a crucial role of HSP90A in a tumor-intrinsic resistance to CTL. Besides immune resistance, we reported the P3 cells have multi-modal resistance to chemotherapy and radiotherapy, and cancer stem cell (CSC)-like property^8,23,24^. Notably, siHSP90AA1 transfection re-sensitized P3 cells to chemotherapy and radiotherapy (Fig. 1d, e). In addition, knockdown of HSP90AA1 in P3 cells decreased in vitro sphere-forming capacity (Fig. 1f and Supplementary Fig. 4) and reduced tumor-initiating property when transplanted into NOD/SCID mice (Table 1), indicating that HSP90A is important for maintenance of CSC-like properties in immune-edited P3 tumor cells. However, P0 cells transfected with siHSP90AA1 did not significantly alter the susceptibility to CTL, cisplatin, and irradiation as well as sphere-forming capacity (Fig. 1c–f). We next investigated whether, as with CaSki P3 model, up-regulation of HSP90A is reproduced in other immune-edited tumor models, TC-1 P3 and MDA-MB231 P3, which were generated from its immune-susceptible parental cell line (TC-1 and MDA-MB231, respectively) through three rounds of in vivo selection by cognate CTLs, respectively^25,26^. Consistent with results from CaSki, we found that mRNA expression of Hsp90aa1 and HSP90AA1 was up-regulated in TC-1 and MDA-MB231 P3 cells, respectively, compared with their parental P0 cells (Supplementary Fig. 5a). We also observed increase of HSP90A protein in both murine and human tumor cells over the course of immunoediting (Supplementary Fig. 5b). Furthermore, we previously reported the both TC-1 P3 and MDA-MB231 P3 cells have multi-modal resistance to immunotherapy and chemotherapy, and CSC-like property^8,25^. Notably, siHsp90aa1 or siHSP90AA1 transfection re-sensitized P3 cells to immunotherapy and chemotherapy, and reduced a stem-like phenotype (Supplementary Fig. 5c–e). Taken together, our data demonstrated that HSP90A is up-regulated at the transcriptional level during immunoediting, and contributes to multi-modal resistance and CSC-like property of immune-edited tumor cells.

**Table 1:** TIC frequency of CaSki P3-no insert, CaSki P3-shHSP90AA1 #1, and CaSki P3-shHSP90AA1 #2 cells^a^.

CaSki P3	Number of tumors/number of injected animals^b^			TIC frequency (95% CI)^c^
	10^5^	10^4^	10^3^	
No insert	6/6	6/6	4/6	1:910 (1/2545–1/326)
shHSP90AA1 #1	4/6	2/6	0/6	1:68,078* (1/166,908–1/27,768)
shHSP90AA1 #2	3/6	2/6	0/6	1:96,052** (1/249,831–1/36,929)

*TIC* tumor-initiating cell, *CI* confidence interval

*p = 4.6 × 10^−10^ and **p = 2.35 × 10^−11^ by pairwise tests

^a^To estimate tumor initiating properties of these cells, in vivo limiting dilution transplantation assay was performed

^b^Tumor cells of indicated number were subcutaneously injected into NOD/SCID mice (*n* = 6) and mice were scored for the presence of palpable tumors

^c^TIC frequency and 95% CI were calculated by using ELDA software program (http://bioinf.wehi.edu.au/software/elda/)

### HSP90AA1 is directly regulated by NANOG

**Fig. 2:**
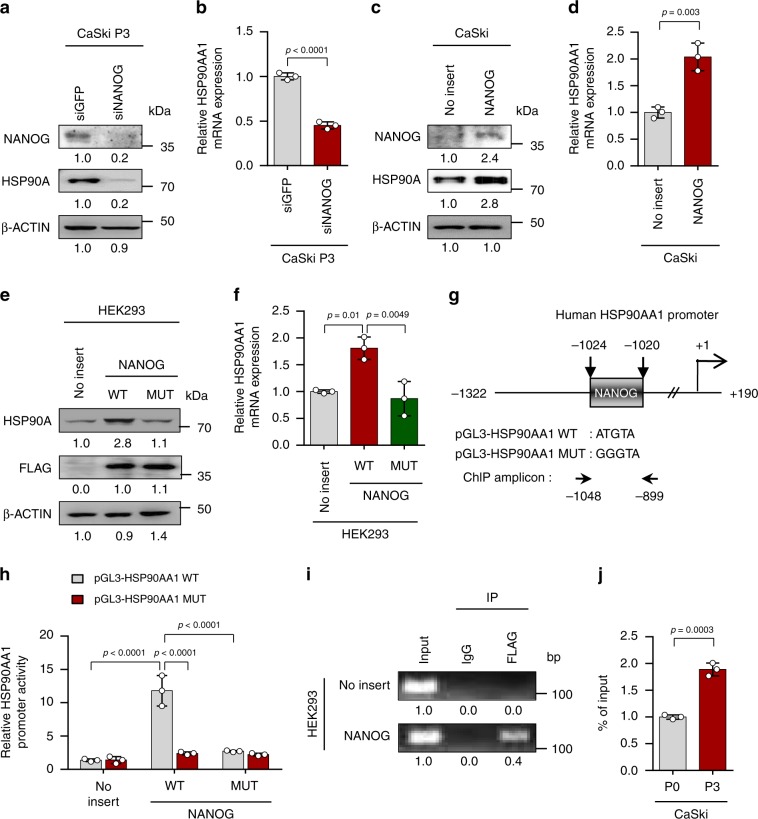
HSP90AA1 expression is directly regulated by NANOG. **a and b** CaSki P3 cells were transfected with siRNA-targeting GFP or NANOG. **a** Levels of NANOG and HSP90A protein were analyzed by Western blot. **b** HSP90AA1 mRNA expression was analyzed by qRT-PCR. **c and d** CaSki P0 cells were stably transfected with empty vector (no insert) or NANOG. **c** Levels of NANOG and HSP90A protein were analyzed by Western blot. **d** HSP90AA1 mRNA expression was analyzed by qRT-PCR. **e** and **f** HEK293 cells were transfected with empty vector (no insert), FLAG-NANOG wild type (NANOG WT) or FLAG-NANOG mutant (NANOG MUT). **e** Levels of HSP90A and FLAG-NANOG proteins were proved by Western blot. **f** HSP90AA1 mRNA expression was analyzed by qRT-PCR. **g** Diagram of HSP90AA1 promoter region (−1322 to +190) containing NANOG binding element. The arrows indicate ChIP amplicon corresponding to −1048 to −899. **h** Luciferase assay in HEK293 cells transfected with the pGL3-HSP90AA1 WT or MUT plasmid, together no insert, NANOG WT or NANOG MUT plasmids. **i** Chromatin immunoprecipitation assay was carried out using HEK293 cells transfected with FLAG-NANOG. Cross-linked chromatin was immunoprecipitated with anti-FLAG antibodies. Immunoprecipitated DNAs were amplified with PCR primers specific for the HSP90AA1 promoter region indicated above. Mouse IgG was used as a negative control. The input represents 2% of the total chromatin. **j** ChIP assay was carried out using CaSki P0 and P3 cells. Cross-linked chromatin was immunoprecipitated with anti-NANOG antibodies. The value of ChIP data represent relative ratio to the input. β-ACTIN was included as an internal loading control. Numbers below blot images indicate the expression as measured by fold change **a**, **c**, and **e**. All experiments were performed in triplicate. The *p*-values by two-tailed Student’s *t* test **b**, **d** and **j**, one-way ANOVA **f** or two-way ANOVA **h** are indicated. Data represent the mean ± SD. Source data are provided as a Source Data file.

We next attempted to elucidate the underlying mechanism responsible for HSP90AA1 up-regulation in immune-edited tumor. In this regards, we previously demonstrated that NANOG is a key TF driving multi-modal resistance and stem-like phenotype of the immune-refractory tumor^25^. Therefore, we hypothesized that NANOG might be responsible for transcriptional activation of HSP90AA1 gene. Indeed, silencing of NANOG in P3 cells resulted in decrease of HSP90A protein, which was accompanied by decreased HSP90AA1 mRNA expression (Fig. 2a, b). Conversely, introduction of NANOG into P0 cells raised HSP90A protein level and HSP90AA1 mRNA expression level (Fig. 2c, d). Notably, NANOG WT profoundly increased levels of HSP90AA1 mRNA and HSP90A protein, while NANOG MUT which was previously characterized for its weak transcriptional activity^16^, had no significant impact on both of HSP90AA1 mRNA and HSP90A protein levels, indicating that NANOG regulates HSP90AA1 expression through its transcriptional function (Fig. 2e, f). To further elucidate the underlying mechanism by which NANOG regulates HSP90AA1 transcription, we identified the HSP90AA1 promoter region containing a putative NANOG-binding site, suggesting the possibility that NANOG is a direct transcriptional activator of HSP90AA1 (Fig. 2g). Luciferase assays showed a significant increase in HSP90AA1 promoter activity upon co-transfection with NANOG WT but not upon co-transfection with NANOG MUT (Fig. 2h). Moreover, mutation of the NANOG-binding site in the HSP90AA1 promoter region eliminated the promoter activation by NANOG WT (Fig. 2h). Chromatin immunoprecipitation (ChIP) assays confirmed the direct binding of NANOG to the regulatory region of HSP90AA1 gene (Fig. 2i), and also validated in the P0 and P3 cells, where we noted more NANOG occupancy in P3 cells, relative to P0 cells (Fig. 2j). Altogether, these findings demonstrate that NANOG up-regulates HSP90AA1 transcription by directly binding to its promoter region.

We then wondered if HSP90A is required for promoting multi-aggressive phenotypes that is mediated by NANOG. Consistently, in the NANOG-overexpressing CaSki-NANOG cells, HSP90AA1 knockdown increased susceptibility to granzyme B, cisplatin, and irradiation (Supplementary Fig. 6a–c) and decreased CSC-like property (Supplementary Fig. 6d). These results indicate that HSP90A plays a crucial role in the NANOG-mediated multi-aggressive phenotypes including immune-refractoriness.

### NANOG–HSP90A axis is conserved across various cancer types

**Fig. 3:**
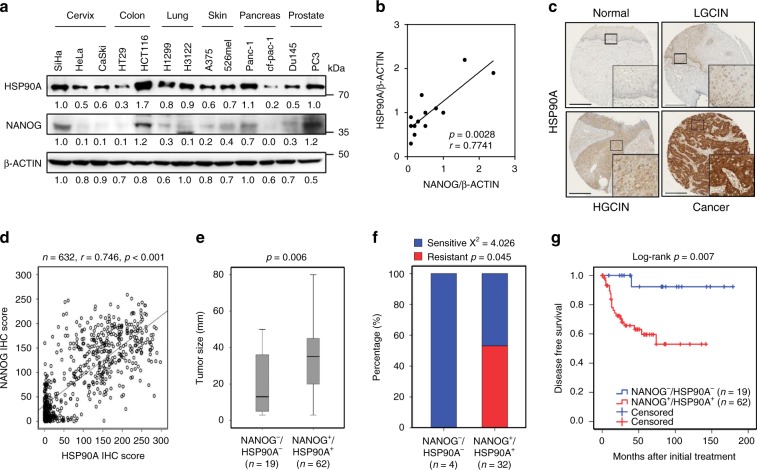
NANOG–HSP90A axis is conserved across various human cancer types. **a** Protein levels of NANOG and HSP90A in various human cancer cells were determined by immunoblotting. This experiment was performed in triplicate. **b** Correlation between NANOG and HSP90A level normalized by β-ACTIN level in various human cancer cells (Spearnan *r* = 0.7741, *p* = 0.0028). **c** Representative images of IHC staining of HSP90A in cervical tissue from normal (*n* = 328), LGCIN (*n* = 65), HGCIN (*n* = 160), and cervical carcinoma (*n* = 151) patients. Scale bar shown is 250 µm. LGCIN low-grade CIN; HGCIN high-grade CIN. **d** Correlation between NANOG and HSP90A in patients with cervical cancer (Spearnan *r* = 0.746, *p* < 0.001). **e** and **f** Combined level of NANOG^+^/HSP90A^+^ was significantly associated with large-sized tumors **e** and chemoradiation resistance **f** in patients with cervical cancer. Chemoradiation resistance was calculated only cases with available information of chemoradiation response. **g** Patients with NANOG^+^/HSP90A^+^ level displayed worse disease-free survival (*p* = 0.007) than patients with NANOG^−^/HSP90A^−^ level. Cut-off value of NANOG^+^ and HSP90A^+^ are 160 and 127, respectively. The *p*-values were determined by Mann–Whitney *U* test **e** and **f** and Log-rank (Mantel–Cox) test **g** or spearman correlation (*r*) **b** and **d**. In the box plots, the top and bottom edges of boxes indicate the first and third quartiles, respectively; the center lines indicate the medians; and the ends of whiskers indicate the maximum and minimum values, respectively. Source data are provided as a Source Data file.

Having explored the molecular mechanism by which the NANOG–HSP90A axis confers tumor-aggressive phenotypes, we examined whether the NANOG–HSP90A axis is conserved across multiple human cancer types. We observed a positive correlation between NANOG and HSP90A protein levels in a variety of human cancer cells (Fig. 3a, b). We then determined the clinical relevance of the NANOG–HSP90A axis in human cancer patients. Comparative transcriptome analysis using the cancer genome atlas (TCGA) data reveals a positive correlation between NANOG and HSP90AA1 mRNA levels in multiple human cancer types, such as cholangiocarcinoma, testicular germ cell tumors, uveal melanoma (Supplementary Fig. 7). Furthermore, we previously had reported that high level of NANOG correlated with poor prognosis of cervical carcinoma^16^. Thus, we evaluated HSP90A protein level by immunohistochemistry in the same study population (Fig. 3d), and found that HSP90A level increased during cervical carcinoma progression (Supplementary Table 1). Upon the assessment between the levels of NANOG and HSP90A in the cervical neoplasia specimens, HSP90A level was positively correlated with that of NANOG (Fig. 3d). Importantly, patients with combined NANOG^+^/HSP90A^+^ level was strongly associated with large-sized tumor (Fig. 3e and Supplementary Fig. 8) and chemo-radiation resistance (Fig. 3f and Supplementary Fig. 9) than those with NANOG^−^/HSP90A^−^ level. In addition, examining the relationship of combined NANOG^+^/HSP90A^+^ level with patient’s survival outcomes, the Kaplan–Meier plots demonstrated that NANOG^+^/HSP90A^+^ patients had shorter disease-free survival than NANOG^−^/HSP90A^−^ patients (Fig. 3g and Supplementary Fig. 10). Consistently, NANOG^+^/HSP90A^+^ patients significantly worse 10-year overall survival than NANOG^−^/HSP90A^−^ patients (Supplementary Fig. 11). Furthermore, the level of NANOG^+^/HSP90A^+^ was a significant risk factor for both disease-free survival (Supplementary Table 2) and overall survival (Supplementary Table 3). Collectively, these data indicate that the NANOG–HSP90A axis is conserved across multiple human cancer types, highly related with therapeutic resistance and an important prognostic factor in human cervical neoplasia.

### HSP90A stabilizes TCL1A, thereby contributing AKT activation

**Fig. 4:**
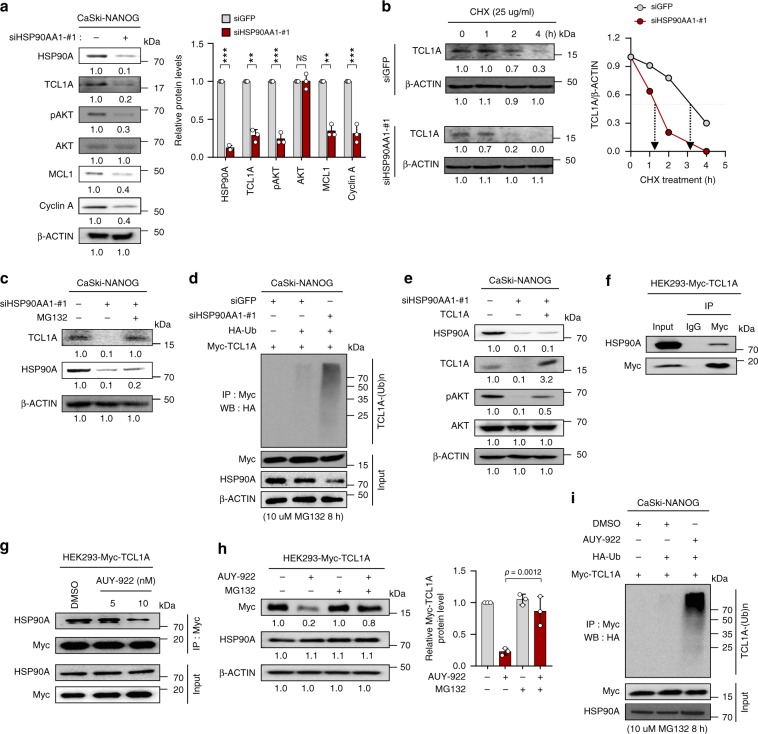
HSP90A contributes to NANOG-induced AKT activation through TCL1A-stabilization. **a–e** CaSki-NANOG cells were transfected with siGFP or siHSP90AA1-#1. **a** Levels of HSP90A, TCL1A, pAKT, AKT, MCL1, and Cyclin A were analyzed by immunoblotting. Graph depicts the experimental quantitation based on at least three independent experiments. **p < 0.001, ***p < 0.0001 by two-tailed Student’s *t* test. NS not significant. **b** The cells were treated with cycloheximide (CHX) for the indicated times. Cell lysates were subjected to immunoblotting with anti-TCL1A antibodies. Graph represents the means ± SD of three quantified data, after normalization to the corresponding β‐ACTIN level. **c** The cells were treated with or without MG132 (10 μM) for 8 h. Cell lysates were subjected to immunoblotting with anti-HSP90A and anti-TCL1A antibodies. **d** The cells, transfecte**d** with indicated plasmids, were treated with MG132. Cell lysates were immunoprecipitated with anti-Myc antibody, and immunoblotted with the anti-HA antibody. The input represents 5% of total proteins. **e** siHSP90AA1-transfect**e**d CaSki-NANOG cells were transfected with TCL1A-Myc constructs. Levels of HSP90A, TCL1A, pAKT, and AKT were analyzed by immunoblotting. **f** HEK293 cells were transfected with TCL1A-Myc. **g** TCL1A-Myc-transfected HEK293 cells were treated with DMSO or AUY-922 as indicated. **f** and **g** Cell lysates were immunoprecipitated with anti-Myc antibody, followed by western blotting using anti-Myc and anti-HSP90A antibodies. **h** HEK293 cells, transfected with indicated plasmids, were treated with or without MG132 (10 μM) for 8 h and were subjected to immunoblotting with anti-Myc and anti-HSP90A antibodies. Graph depicts the experimental quantitation of TCL1A protein level based on at least three independent experiments. The *p*-value by one-way ANOVA is indicated. **i** CaSki-NANOG cells were transfected with HA-Ub or TCL1A-Myc constructs and then were treated with DMSO or AUY-922 as indicated. Cell lysates were immunoprecipitated with anti-Myc antibody, and immunoblotted with the anti-HA antibody. The input represents 5% of total proteins. Numbers below blot images indicate the expression as measured by fold change **a**–**c**, **e** and **h**. All experiments were performed in triplicate. Data represent the mean ± SD **a** and **h**. Source data are provided as a Source Data file.

Previously, we demonstrated that NANOG promotes tumorigenicity and immune resistance of tumor cells through AKT-dependent up-regulation of Cyclin A and MCL-1 (ref. ^16^). Despite NANOG overexpression, knockdown of HSP90AA1 robustly dampened levels of pAKT, Cyclin A, and MCL-1 (Fig. 4a). Consistently, silencing of HSP90AA1 in P3 cells but not in P0 cells (Supplementary Fig. 12a) and NANOG up-regulated human cancer cells (Supplementary Fig. 12b) markedly reduced the levels of them. Importantly, overexpression of constitutively active AKT (CA-AKT) decreased the sensitivity of siHSP90AA1-transfected CaSki-NANOG cells to CTLs and cisplatin (Supplementary Fig. 13), suggesting that HSP90A plays a crucial role in NANOG-mediated phenotypes by reinforcing the link between NANOG and AKT-signaling pathway.

We next aimed to elucidate the role of HSP90A in NANOG-induced activation of AKT signaling. In this regard, we previously demonstrated that NANOG hyper-activates the AKT signaling through transcriptional up-regulation of TCL1A, a co-activator of AKT kinase^27^. Given the role of HSP90A as a chaperone in protein stabilization, we questioned whether HSP90A affects protein levels of AKT or TCL1A. The down-regulation of pAKT level upon HSP90AA1 knockdown, however, was not accompanied with loss of AKT protein (Fig. 4a and Supplementary Fig. 12). In contrast, knockdown of HSP90AA1 in CaSki-NANOG cells significantly decreased the levels of TCL1A protein (Fig. 4a). To assess whether HSP90A affects TCL1A stability, we measured the half-life of TCL1A protein upon HSP90AA1 knockdown by performing a cycloheximide-chase assay. Notably, the half-life of endogenous TCL1A protein in siHSP90AA1-transfected CaSki-NANOG cells decreased, compared with those of the siGFP-transfected cells (Fig. 4b). In addition, treatment of MG132 blocked TCL1A protein down-regulation upon HSP90AA1 knockdown (Fig. 4c), indicating the proteasome-mediated degradation of TCL1A protein. Since poly-ubiquitination is required for proteasome-dependent protein degradation, we examined whether HSP90A affects ubiquitination of TCL1A protein. As shown in Fig. 4d, ubiquitination of TCL1A was significantly increased by HSP90AA1 knockdown. Notably, we noted that decreased pAKT level and increased susceptibility to CTLs and cisplatin after HSP90AA1 knockdown in CaSki-NANOG cells were reversed upon restoration of TCL1A expression (Fig. 4e and Supplementary Fig. 14). These results suggest that HSP90A-mediated TCL1A stabilization is important for NANOG-induced AKT activation as well as multi-modal resistance.

We then assessed the effect of HSP90AA1 overexpression on CaSki P0 cells. Overexpression of HSP90AA1 in P0 cells increased TCL1A stability and pAKT level (Supplementary Fig. 15a and b). Importantly, HSP90AA1-transfected P0 cells were more resistant to apoptosis induced by CTLs, compared to empty vector-transfected P0 cells (Supplementary Fig. 15c), indicating that HSP90AA1 expression by itself is sufficient to confer resistance to CTLs. Taken together, our data indicate that HSP90A leads to activation of AKT signaling through TCL1A stabilization, and thus contributes to multi-modal resistance of tumor cells.

Previously, it was reported that chemical inhibition of HSP90 promotes the proteosomal degradation of HSP90 client proteins by inhibiting its chaperone association^28^. Therefore, we determined whether TCL1A has chaperone association with HSP90A, as a client protein. Notably, TCL1A protein exhibited chaperone associated with HSP90A (Fig. 4f), which was disrupted after treatment with AUY922, the HSP90A inhibitor (Fig. 4g). Consistently, treatment with AUY922 also promotes ubiquitin-dependent proteosomal degradation of TCL1A protein (Fig. 4h, i). Furthermore, HSP90A level was positively correlated with TCL1A level in the cervical neoplasia specimens (Supplementary Fig. 16a) and patients with combined HSP90A^+^/TCL1A^+^ level exhibited a tendency of worse disease-free survival (Supplementary Fig. 16b–e). Thus, our results demonstrate that TCL1A is a client protein of HSP90A, and that HSP90A’s direct binding leads to it stabilization, thereby contributing to AKT activation in NANOG^high^ tumor cells.

### HSP90A is a therapeutic target for NANOG^high^ tumor cells

**Fig. 5:**
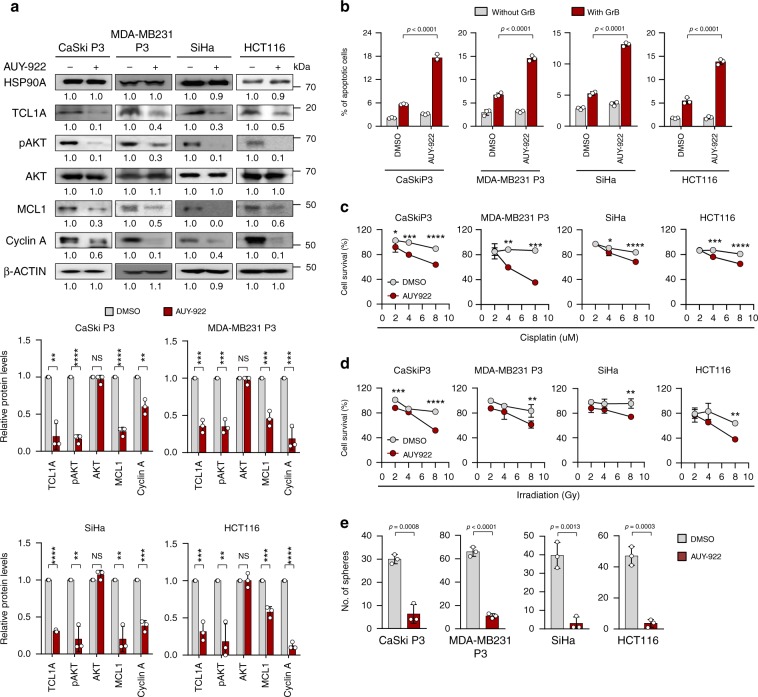
HSP90A inhibition reduces multi-aggressive properties of NANOG^high^ tumor cells. **a–e** CaSki P3, MDA-MB231 P3, SiHa, and HCT116 cells were treated with DMSO or AUY-922. **a** Levels of HSP90A, TCL1A, pAKT, AKT, MCL1, Cyclin A, and β‐ACTIN were proved by Western blot. β-ACTIN was included as an internal loading control. Numbers below blot images indicate the expression as measured by fold change. Graph depicts the experimental quantitation based on at least three independent experiments. **b** Flow cytometry analysis of the frequency of apoptotic (active caspase-3^+^) cells in the cells after intracellular delivery of granzyme B for 4 h. **c** and **d** Cells were treated with indicated concentrations of cisplatin **c** and irradiation **d**. The percentage of viable cells was determined by trypan blue exclusion assay at 24 h after either drug challenge or irradiation. **e** Sphere-forming capacity of the cells in low-density suspension culture. All experiments were performed in triplicate. The *p*-value by two-tailed Student’s *t* test **a**, one-way ANOVA **b** and **e** or two-way ANOVA **c** and **d** are indicated. *p < 0.05, **p < 0.01, ***p < 0.001, ****p < 0.0001. Data represent the mean ± SD. Source data are provided as a Source Data file.

To verify the biochemical effects of HSP90A–TCL1A–AKT axis in diverse types of human cancer cells, we further selected two NANOG up-regulated human cancer cells SiHa and HCT116, and immune-edited MDA-MB231 P3 (ref. ^25^). Inhibition of HSP90A with AUY922 robustly dampened AKT phosphorylation level and expression of the effectors in NANOG signaling, such as MCL-1, Cyclin A, and TCL1A across all tested cancer cells (Fig. 5a). Furthermore, AUY922-treated tumor cells were more susceptible to multi-modal therapy and showed diminished sphere-shaping capacities compared with DMSO-treated tumor cells (Fig. 5b–e). These results demonstrate that the functional and biochemical properties of the HSP90A–TCL1A–AKT axis are conserved across multiple types of cancer cells and that HSP90A is an actionable target for controlling NANOG^high^ human tumor cells.

### HSP90A inhibition reverses resistance to ACT

**Fig. 6:**
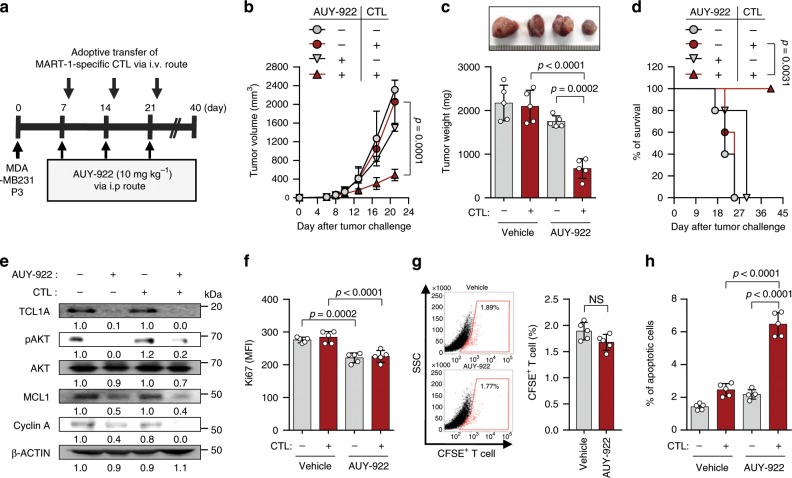
HSP90A inhibition reverses the resistant phenotype to adoptive T cell therapy. **a** Schematic of the therapy regimen in NOD-SCID mice implanted with MDA-MB231 P3 cells. **b** Tumor growth, **c** mass (at 21 days after challenge), and **d** survival of mice inoculated with MDA-MB231 P3 treated with the indicated reagents. **e** Western blot analysis of TCL1A, pAKT, AKT, MCL1, Cyclin A protein in mice administered with PBS or AUY-922, with or without adoptive transfer of MART-1-specific CTLs. β-ACTIN was included as an internal loading control. Numbers below blot images indicate the expression as measured by fold change. **f** The proliferation index of cells inside the tumor, as measured by the mean fluorescence intensity of Ki67 staining. **g** Flow cytometric analysis of the frequency of CFSE-labeled MART-1-specific CTL in the tumors of mice that received adoptive transfer. **h** The frequency of apoptotic cells in the tumors of PBS-treated or AUY-922-treated mice, with or without adoptive transfer of MART-1–specific CTL. For in vivo experiments, five mice from each group were used. The *p*-value by two-way ANOVA **b**, one-way ANOVA **c**, **f** and **h**, Log-rank (Mantel–Cox) test **d** or two-tailed Student’s *t* test **g** are indicated. Data represent the mean ± SD. Source data are provided as a Source Data file.

Given our observations in vitro, we reasoned that in vivo administration of AUY-922 should reverse resistance to T cell-base immunotherapy. To test this possibility, we treated MART1^+^ MDA-MB231 P3-bearing NOD-SCID mice with MART-1-specific CTLs along with AUY-922 (Fig. 6a). While immunotherapy alone had no effect on tumor growth, dual therapy with E7-specific CTLs and AUY-922 retarded tumor growth (Fig. 6b, c) and prolonged survival of the mice (Fig. 6d). Consistent with our in vitro results, we observed reduced levels of TCL-1, pAKT, MCL-1, and Cyclin A in tumor tissue from AUY922-treated mice compared with that from un-treated mice (Fig. 6e). In addition, measurements by Ki67 staining demonstrated that AUY-922-treated tumors contained fewer proliferating cells than PBS-treated tumors, as this was unaffected by the adoptive transfer of CTL (Fig. 6f). Although we observed a slight, but not statistically significant decrease in the frequency of antigen-specific CTLs in the tumors of AUY-922-treated mice compared with those in PBS-treated mice, the overall cytotoxic effect of these CTLs was greater after treatment of AUY-922 relative to that than PBS control, as indicated by the percentage of apoptotic tumor cells (Fig. 6g, h). Taken together, we conclude that inhibition of HSP90A can incapacitate the immune resistance of immune-edited tumor cells and represents an attractive strategy for the control of human cancer, as a synergistically, as part of a T cell-mediated immunotherapy.

### HSP90AA1 is associated with poor response to ICB

**Fig. 7:**
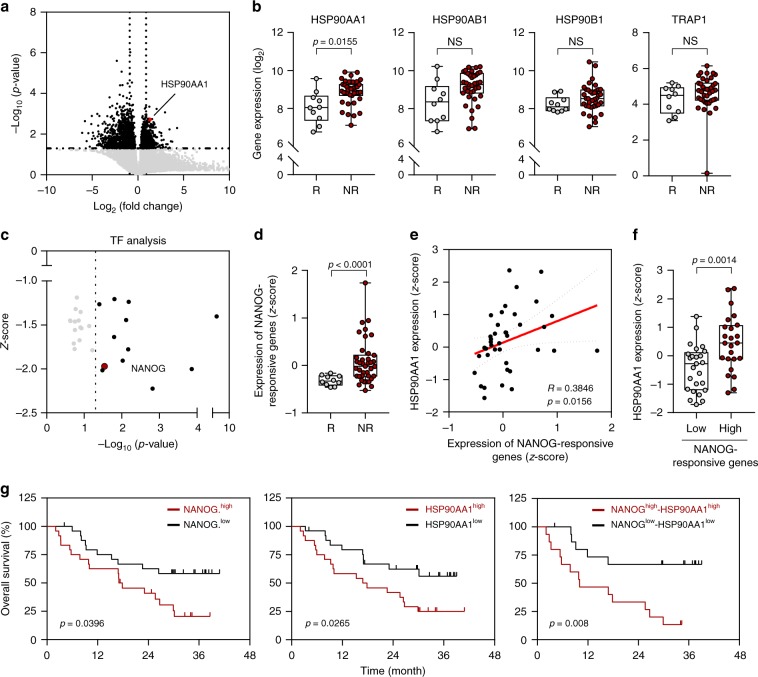
NANOG–HSP90AA1 axis is associated with poor response to PD-1 blockade therapy. **a** Volcano plot shows the DEGs between non-responder and responder to PD-1 blockade therapy. Colors of dots indicate significantly (black) or non‐significantly (gray) altered genes in above or below the horizontal dashed line at *p* = 0.05. Red dot indicates the HSP90AA1 gene. Vertical dashed lines indicate a fold change cut off (1.5FC). The *p*-values were determined by two-tailed test. **b** Comparisons of expressions levels of HSP90AA1, HSP90AB1, HSP90B1, and TRAP1 in responder (R, *n* = 10) and non-responder (NR, *n* = 39). **c** TF analysis of up-regulated DEGs in non-responders relative to responders to anti-PD-1 therapy. Each dot represents one TF which has transcription factor motifs enriched in DEGs. Vertical dashed line indicates *p*-value cutoffs at the 0.05 level. **d** Comparisons of expression level of NANOG-responsive genes in the responder (R, *n* = 10) and non-responder (NR, *n* = 39). **e** Correlation between expression level of NANOG-responsive genes and HSP90AA1 in non-responder (Spearman’s *R* = 0.3846, *p* = 0.0156). **f** Comparisons of HSP90AA1 expression in non-responders with low levels (Low *n* = 18) and high levels (High, *n* = 21) of NANOG-responsive genes. Error bars represent standard deviations from the mean. The *p*-value by unpaired *t*-test are indicated. **g** Kaplan–Meier analysis of overall survival (calculated as months to death or months to last follow-up) and median expression value cutoffs for expression level of NANOG-responsive genes (left, NANOG.^high^ > median; NANOG.^low^ < median, *p* = 0.0396) and HSP90AA1 (HSP90AA1^high^ > median; HSP90AA1^low^ < median, *p* = 0.0265, middle) and combined each (*p* = 0.008, right). The *p*-values were determined by Gehan–Breslow–Wilcoxon test. In the box plots, the top and bottom edges of boxes indicate the first and third quartiles, respectively; the center lines indicate the medians; and the ends of whiskers indicate the maximum and minimum values, respectively. Source data are provided as a Source Data file.

Based on our observations, we further questioned that NANOG–HSP90A axis may also contribute poor response to ICB. To determine the clinical relevance of NANOG–HSP90A axis, we used the transcriptome data from melanoma patients classified as responders (R) or non-responders (NR) to anti-PD-1 therapy^29^. From the comparative transcriptome analysis of differentially expressed genes (DEGs) in two patient groups (Fig. 7a), we found that only the HSP90AA1 expression level among the four genes of the HSP90 family (HSP90AA1, HSP90AB1, HSP90B1, TRAP1) was significantly higher in NR compared with R (Fig. 7b).

It has been reported that multi-gene signature is associated with clinical efficacy of PD-1 blockade^30^. To gain insights into NANOG as a TF responsible for response to anti-PD-1 therapy, we performed TF analysis using up-regulated or down-regulated DEGs in NR relative to R to anti-PD-1 therapy. From this analysis, we noted that many of the up-regulated DEGs in NR were directly linked to NANOG (Fig. 7c). On the other hand, genes regulated by IRF8, a TF in the interferon gamma (IFN-γ)-signaling pathway, were down-regulated in NR to anti-PD-1 therapy (Supplementary Fig. 17a and b). This finding is consistent with recent studies demonstrating that an IFN-γ signature was found to be differentially down-expressed in the pretreatment tumor biopsies from non-responding patients^31–33^.

To validate preliminary NANOG-responsive genes from this analysis, we further performed a complementary analysis followed by filtering to include only genes that have been previously identified as responding to NANOG^34–38^. Subsequently, we acquired the refined NANOG-responsive six genes (FGFBP1, SPESP1, HMGA2, PERP, FGF1, and DKK1). To quantify the expression of NANOG-responsive genes, we calculated the average expression of each gene. Notably, the expression level of the NANOG-responsive genes was significantly higher in NR compared with R to anti-PD-1 therapy (Fig. 7d). Furthermore, we also found a strong correlation between NANOG-responsive genes and HSP90AA1 expression in NR (Fig. 7e, f).

We next examined the clinical relevance of the NANOG–HSP90A axis. Kaplan–Meier plots demonstrated that patients with high levels of NANOG-responsive genes or HSP90AA1 displayed worse overall survival rate (Fig. 7g). Furthermore, patients with high expression levels of both NANOG-responsive genes and HSP90AA1 showed significantly worse overall survival rate (Fig. 7g). Taken together, our data suggest that NANOG–HSP90AA1 axis could be a biomarker in predicting response and clinical outcome to anti-PD-1 therapy, and provide a rationale for the clinical use of HSP90A inhibitors.

### HSP90A inhibition renders tumor susceptible to PD-1 blockade

We next assessed whether NANOG–HSP90A axis would be also responsible for refractoriness to ICB therapy. To do this, we examined levels of NANOG and HSP90A in a number of mouse cancer cell lines, and then selected a murine tumor cell line having high level of both NANOG and HSP90A, B16-F10, which is relatively resistant to ICB therapy^39^ (Supplementary Fig. 18a). Knockdown of NANOG or inhibition of HSP90A with AUY-922 in B16-F10 cells substantially decreased the levels of TCL1A, pAKT, MCL1, and Cyclin A (Supplementary Fig. 18b and c). Consistently, the AUY-922-treated tumor cells were more susceptible to apoptosis induced by granzyme B compared to DMSO-treated tumor cells (Supplementary Fig. 18d). These data indicate that biochemical and functional properties of the NANOG–HSP90A–TCL1A axis are conserved in the immune-refractory murine tumor model.

**Fig. 8:**
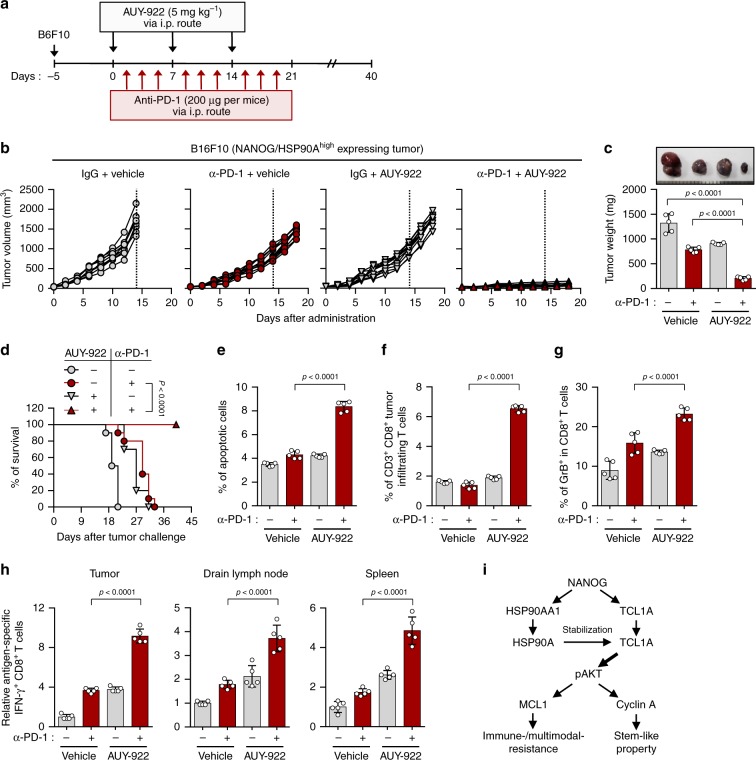
HSP90A inhibition renders the tumor susceptible to anti-PD-1-mediated anti-tumor immunity. **a** Schematic of the therapy regimen in mice implanted with B16F10 cells. **b–h** Tumor-bearing mice administered vehicle or AUY-922, with or without treatment of PD-1 antibody. **b** Tumor growth and **c** tumor mass in mice at 19 days after challenge. **d** Survival of mice inoculated with B16F10 cells treated with the indicated reagents. **e** The frequency of apoptotic cells in the tumors of vehicle-treated or AUY-922-treated mice, with or without anti-PD-1 treatment. **f** Flow cytometry profiles of tumor-inflitrating CD3^+^ and CD8^+^ T cells. **g** The ratio of Granzyme B^+^ to tumor-inflitrating CD3^+^ CD8^+^ T cells. **h** Quantification of antigen-specific CTLs in tumor (left), drain lymph node (middle), or spleen (right) derived from the tumor-bearing mice. **i** Schematic interpretation of the molecular mechanism by which NANOG–HSP90A–TCL1A axis promotes multi-modal-resistance and stem-like property of immune-refractory tumor cells. Numbers below blots indicate the expression as measured by fold change. For in vivo experiments, 10 mice from each group were used. The *p*-value by one-way ANOVA **c** and **e**–**h** or Log-rank (Mantel–Cox) test **d** is indicated. Data represent the mean ± SD. Source data are provided as a Source Data file.

Responses to ICB therapy in patients is predictable by pre-existing CD8^+^ T cells that can most robustly be measured via expression of CD8^+^ T cell signature^11,40^. Interestingly, we found that level of NANOG-responsive genes and HSP90AA1 within tumor inversely correlated with T cell infiltration in various cancer patients from TCGA (Supplementary Fig. 19), suggesting NANOG–HSP90A axis in tumor cells induces ICB therapeutic resistance. To investigate whether NANOG–HSP90A axis is responsible for resistance to anti-PD-1 therapy in vivo, we treated NANOG/HSP90A^high^ (B16F10 or MC38) or NANOG/HSP90A^low^ (B16F1 or CT26) tumor-bearing mice with anti-PD-1 antibody along with AUY-922 (Fig. 8 and Supplementary Fig. 20). Anti-PD-1 antibody alone treatment showed a remarkable therapeutic effect in NANOG/HSP90A^low^ (B16F1 and CT26) tumor-bearing mice (Supplementary Fig. 20b and c). In contrast, in NANOG/HSP90A^high^ (B16F10 or MC38) tumor-bearing mice, while anti-PD-1 antibody alone treatment had no effect on tumor growth, the combination treatment with anti-PD-1 antibody and AUY-922 retarded tumor growth (Fig. 8b, c and Supplementary Fig. 20d) and prolonged survival of tumor-bearing mice (Fig. 8d). These results suggest that NANOG–HSP90A axis could be responsible for resistance to anti-PD-1 therapy, and HSP90A inhibition overcomes NANOG–HSP90A axis-mediated resistant phenotype of tumor cells to immunotherapy.

Accumulating evidence suggest that tumor cell death in anti-tumor therapy could lead to release of tumor antigens that prime subsequent auto-loop of anti-tumor immune responses (known as the cancer-immunity cycle)^12–14^. We found that the percentage of apoptotic tumor cells was increased in the combination treatment compared with either treatment alone (Fig. 8e). Therefore, we postulated that HSP90A inhibition along with anti-PD-1 treatments inflames tumor immune-environments by reversing resistance of immune-refractory tumor cells to T-cell-mediated cytotoxicity. Indeed, the number of overall CD8^+^ T cells and tumor-reactive CD8^+^ T cells making granzyme B were significantly higher in the combination treatment group compared to the other treatment groups (Fig. 8f, g), indicating that the infiltration of tumor-killing CD8^+^ T cells could be enhanced by the combined treatment. To test whether the generation of tumor-reactive T cells was affected by dual treatment, we isolated cells from tumor, drain lymph node, or spleen of mice received therapy, stimulated ex vivo with either DMSO (control) or gp100 (B16-F10 tumor-associated antigen), and then assessed IFN-γ producing CD8^+^ T cells. Notably, IFN-γ-positive CD8^+^ T cells following stimulation with gp100 were increased in combination treatment group compared to the other treatment groups (Fig. 8h). Taken together, our results demonstrate that the HSP90A inhibition enhances response to ICB therapy by reversing immune resistance of tumor cells to CTL killing, which switches immune phenotypes from non-T cell inflamed to T cell-inflamed tumors.

## Discussion

Immunoediting of tumor cells can provoke multi-modal resistance to chemotherapy, radiotherapy, as well as immunotherapy and promote a stem-like phenotype. In the process, NANOG mediates the pro-survival and stem-like phenotype of the immune-edited tumor cells through the AKT pathway. Thus, strategies impeding the NANOG-signaling pathway may not only conquer the problem of therapeutic resistance but also that of the stem-like state in cancer. In this study, we demonstrated the crucial role of HSP90A at the crossroads between NANOG signaling and the multi-aggressive properties of immune-edited tumor cells by identifying HSP90AA1 as a NANOG transcriptional target. In this vein, the NANOG–HSP90A axis was widely conserved in various TCGA cohorts and tumor cells derived from multiple types of human cancer, as well as in tumor tissue from patients with cervical cancer. Furthermore, levels of NANOG and HSP90A within the tumor correlated strongly with disease progression and survival in cancer patients, indicating that the expression status of HSP90A (either alone or in conjunction with NANOG) within tumor tissue may serve as an excellent prognostic marker.

As the NANOG–HSP90A axis has been implicated as a central channel in the development of immune-resistant phenotypes, we believe that inhibition of HSP90A may be an effective strategy to control immune-refractory tumor cells. Indeed, HSP90A inhibition has received attention for therapeutic purposes in solid tumors and hematologic malignancies, even though it has shown limited responses as a single agent in cancer patients. In this study, we provide rational for combination of HSP90A inhibition with T cell-mediated immunotherapy. Notably, HSP90A inhibition with AUY-922 in tumor cells following immunoediting restores sensitivity to lysis by CTLs and effectively controls tumor growth in mice transferred with tumor-specific CTLs. Furthermore, AUY-922 treatment enhances anti-PD-1 therapy in B16F10 tumors, which have no significant response to anti-PD-1 therapy. To extend our findings to clinical relevance, we examined clinical samples from patients treated with anti-PD-1 antibody and noted an association between increased expression of HSP90AA1 and non-response to anti-PD-1 therapy, suggesting the crucial role of HSP90A in resistance to anti-PD-1 therapy as well as ACT.

Given the crucial role of HSP90A in intrinsic resistance to CTLs, it is intriguing, and perhaps counterintuitive, that the combination of AUY-922 and anti-PD-1 therapy further increase trafficking of effector CD8^+^ T cells to tumors. Nonetheless, it has been documented that tumor cell death in anti-tumor therapy may lead to release of tumor antigens that prime subsequent immune responses, known as the cancer-immunity cycle. The cancer-immunity cycle tightly modulates immune phenotypes in the tumor microenvironment^41,42^. All human cancers can be grouped into two immune profiles, non-T cell inflamed and T cell inflamed, depending on their immune phenotypes^43^. Accumulating evidence suggest that disruption of one or more steps of the cancer-immunity cycle may trigger the switch of immune phenotypes from T cell-inflamed tumor to non-T cell-inflamed tumor^10,42–45^. Particularly, T cell-mediated killing of tumor cells is important for initiating or re-invigorating the cancer-immunity cycle by providing stimuli of tumor antigens including neo-antigens to cognate T cells^13,14^. These studies suggest that intrinsic resistance to CTLs could be a crucial cause to disrupt a self-sustaining cancer-immunity cycle that determines immune phenotypes. In light of this, our results demonstrate that HSP90A inhibition could induce T cell-inflamed immune phenotypes by reversing resistance to T-cell-mediated cytotoxicity in tumor microenvironments, indicating the re-invigoration of the cancer-immunity cycle.

Notably, HSP90A is important in maintenance of structural integrity for its client proteins, thereby regulating a variety of cellular processes^18^. In this regard, many of its client proteins are known oncogenic drivers that can regulate tumor intrinsic pathways, some of which may provide a route of interference in response to immunotherapy^46^. Here, we discovered that HSPA90A plays a crucial role in NANOG-mediated phenotypes by reinforcing the link between NANOG and the AKT-signaling pathway. In the course of elucidating HSP90A’s function in NANOG signaling, we identified TCL1A as a client protein of HSP90A. With the reports on how TCL1A activates the AKT pathway as an adaptor that facilitates AKT dimerization and cross phosphorylation^27^, we found that NANOG cooperatively reinforces TCL1A-AKT signaling, a key pathway in multi-aggressiveness of immune-refractory tumor cells by up-regulation of HSP90A (Fig. 8i). Although AKT has been identified as one of HSP90’s client proteins^47^, it seems that dampening of the AKT signal through HSP90A inhibition is not due to the loss of AKT proteins. In our present study, we demonstrate that HSP90A potentiates the NANOG-induced AKT activation through TCL1A stabilization, thereby contributing to the NANOG-mediated multi-malignant phenotypes.

It should be noted that the roles of extracellular HSP90A in mediating tumor progression or promoting anti-tumor immunity remains a complex and controversial matter. Notably, it is obvious that surface-localized or extracellularly released HSP90 in situations not involving cell death might also benefit the tumor^48^. For instance, extracellular HSP90A plays a role in sustaining cancer cell motility, invasion, and metastatic spread^20,49–51^. Nevertheless, it has been well documented that cell surface and extracellular HSPs are most commonly associated with induction of immune responses^52^. Indeed, surface exposure of HSP90 induced by antitumor drugs on dying myeloma cells promotes the induction of specific immune responses to tumor cells^52^. In light of this, we postulate that intracellular HSP90A could serve as extracellular HSPs, potentially leading to anti-tumor immunity, if the tumor cells are dying or exposed to cellular stress in tumor microenvironment.

In general, HSP90s act as molecular chaperones in concert with other chaperones including HSP70 to provide maturation and folding of their client proteins^53^. Indeed, HSP90 and HSP70 have been linked to cancer resistance to stress-mediated apoptotic signals^54^. Paradoxically, HSP90 inhibition with AUY-922 consistently leads to up-regulation of HSP70 inside and outside of tumor cells^55^. The induction of HSP70 may ultimately limit the efficacy of HSP90 inhibitors under certain circumstances^54^. Nonetheless, it has been well documented that tumor-derived extracellular HSP70 could contribute to activation of antitumor immunity by acting as damage-associated molecular pattern (DAMP) or chemokine^56,57^. In addition, cell surface-bound HSP70 mediates granzyme B-dependent apoptosis by specific binding and uptake of granzyme B^58^. Therefore, it will be important in future studies to assess involvement of other chaperones including HSP70 in promoting anti-tumor immunity to AUY922.

Altogether, we propose that NANOG^+^ cancer cells enriched by immunoediting drive preferential expression of HSP90AA1 via transcriptional regulation and undergo HSP90A accumulation in tumor cells. Through this process, HSP90A potentiates NANOG-induced AKT activation through TCL1A stabilization, solidifying the NANOG-mediated multi-aggressive phenotypes of immune-refractory tumors. Furthermore, we demonstrate that inhibition of HSP90A with AUY-922 enhances tumor susceptibility to T-cell-based immunotherapy by re-invigorating the cancer-immunity cycle. Therefore, our data provide evidence that the inhibition of HSP90A may be a promising strategy that will help combat NANOG^+^ immune-refractory tumors, particularly in regard to immune-based cancer therapy.

## Methods

### Mice

Six- to eight-week-old female NOD/SCID or C57BL/6 mice were purchased from Central Lab. Animal Inc. (Seoul, Korea). All mice were maintained and handled under the protocol approved by the Korea University Institutional Animal Care and Use Committee (KOREA-2017-0141). All animal procedures were performed in accordance with recommendations for the proper use and care of laboratory animals.

### siRNA and shRNA constructs

Synthetic small interfering RNAs (siRNAs) specific for GFP, HSP90AA1, and NANOG were purchased from Bioneer (Korea); Non-specific GFP (green fluorescent protein), 5′-GCAUCAAGGUGAACUUCAA-3′ (sense), 5′-UUGAAGUUCACCUUGAUGC-3′ (antisense); HSP90AA1#1, 5′-CACCAAACAUAACGAUGAU-3′ (sense), 5′-AUCAUCGUUAUGUUUGGUG-3′ (antisense); HSP90AA1#2, 5′-UGAAGGAGAUGACGACACA-3′ (sense), 5′-UGUGUCGUCAUCUCCUUCA-3′ (antisense); HSP90AA1#3, 5′-GGCACCUGUUAACUGGUACCA-3′ (sense), 5′-UGGUACCAGUUAACAGGUGCC-3′ (antisense); NANOG, 5′-GCAACCAGACCUGGAACAA-3′ (sense), 5′-UUGUUCCAGGUCUGGUUGC-3′ (antisense). For stable knockdown of HSP90AA1, the following complementary oligonucleotides were annealed and cloned into BamHI/HindIII-digested pSilencer 3.1-H1 puro vector (Ambion Inc., Austin, TX): shHSP90AA1#1, 5′-GATCCGCACCAAACATAACGATGATTTCAAGAGAATCATCGTTATGTTTGGTGTTTTTTGGAAA-3′ (sense), 5′-AGCTTTTCCAAAAAACACCAAACATAACGATGATTCTCTTGAAATCATCGTTATGTTTGGTGCG-3′ (antisense); shHSP90AA1#2, 5′- GATCCGTGAAGGAGATGACGACACATTCAAGAGATGTGTCGTCATCTCCTTCATTTTTTGGAAA-3′ (sense), 5′-AGCTTTTCCAAAAAATGAAGGAGATGACGACACATCTCTTGAATGTGTCGTCATCTCCTTCACG-3′ (antisense).

### Cell lines and reagents

CaSki, MDA-MB231, SiHa, HCT116, and HEK293 cell lines were purchased from American Type Culture Collection (ATCC, Manassas, VA, USA). All cell lines were obtained between 2010 and 2014, and tested for mycoplasma using Mycoplasma Detection Kit (Thermo Fisher Scientific, San Jose, CA, USA). The identities of cell lines were confirmed by short tandem repeat (STR) profiling by IDEXX Laboratories Inc. and used within 6 months for testing. Generation and maintenance of the immune-edited CaSki P3 (ref. ^22^) and MDA-MB P3 (ref. ^25^) cell lines have been previously described. CaSki-NANOG cell line has been previously described^25^. For generation of stable HSP90AA1 knockdown-CaSki P3 cell lines, pSilencer-shHSP90AA1#1, -shHSP90AA1#2, or empty vector (no insert) were transfected into CaSki P3 cells. Stably transfected cells were selected with puromycin (0.7 μg mL^−1^). All cells were grown at 37 °C in a 5% CO_2_ incubator/humidified chamber. Cisplatin and AUY-922 were purchased from Selleckchem (Huston, TX, USA).

### DNA constructs and site-directed mutagenesis

The pMSCV-NANOG WT and MUT plasmids have been described previously^16^. pME18-myc/TCL1 was kindly provided by Dr. Masayuki Noguchi of Hokkaido University^27^. To generate the pGL3-HSP90AA1 promoter, the promoter region of the HSP90AA1 gene was isolated by PCR from genomic DNA extracted from CaSki cells using the following primer set, 5′-TTCTCGAGTTCACACGCTCTTAAGGGGG-3′ (forward) and 5′-CCAAGCTTCTTGGCTAAGTGACCGCACA-3′ (reverse). The PCR products were digested with XhoI and HindIII and subcloned into the XhoI/HindIII restriction sites of the pGL3-Basic vector (Promega). Site-directed mutagenesis was performed using a QuickChange XL Site-directed Mutagenesis kit (Stratagene, San Diego, CA, USA) according to the manufacturer’s instructions. To create mutations in the NANOG-binding sites of the HSP90AA1 promoter region, the following primer sets were used: for pGL3-HSP90AA1 promoter-NANOG MUT, 5′-CAAGATATCCGAAAATTCCCGGGTAGAAACTGGCTTTTCC-3′ (sense) and 5′-TGGAAAAGCCAGTTTCTACCCGGGAATTTTCGGATATCTTG-3′ (antisense). Mutations were verified by DNA sequencing.

### Real-time quantitative RT-PCR

Total RNA was isolated using RNeasy Micro kit (Qiagen), and the cDNAs were synthesized by reverse transcriptase (RT) using iScript cDNA synthesis kit (Bio-Rad), according to the manufacture’ recommended protocol. Real-time quantitative PCR was performed using iQ SYBR Green super mix (Bio-Rad) with the specific primers on a CFX96 real-time PCR detection system. All real-time quantitative PCR experiments were performed in triplicate and quantification cycle (Cq) values were determined using Bio-Rad CFX96 Manager 3.0 software. Relative quantifications of the mRNA levels was performed using the comparative Ct method with β-ACTIN as the reference gene. The sequences of primers used for real-time PCR experiments are shown in Supplementary Table 4.

### Western blot analysis

Lysate extracted from a total of 1 × 10^5^ cells was used to perform Western blot. Primary antibodies against HSP90A (PA3-013, Thermo Fisher Scientific, Fremont, CA), AKT (#9272, Cell Signaling Technology, Danvers, MA), phospho-AKT (S473) (#9271, Cell Signaling Technology), MCL1 (sc-819, Santa Cruz Biotechnology, CA), Cyclin A (sc-239, Santa Cruz Biotechnology), NANOG (A300-397A, Bethyl Laboratories, Montgomery, TX), FLAG (M185-3L, MBL, Nagoya, Japan), Myc (M192-3, MBL), HA (sc-805, Santa Cruz Biotechnology), and β-ACTIN (M177-3, MBL) were used. Western blotting was followed by the appropriate secondary antibodies conjugated with horseradish peroxidase. Immunoreactive bands were developed with the chemiluminescence ECL detection system (Elpis Biotech, Daejeon, Korea), and signals were detected using a luminescent image analyzer (LAS-4000 Mini, Fujifilm, Tokyo).

### ChIP and quantitative ChIP (qChIP) assays

The ChIP kit (Millipore) was employed according to the manufacturer’s instructions. Briefly, cells (1 × 10^7^ per assay) were bathed in 1% formaldehyde at 25 °C for 10 min for cross-linking of proteins and DNA and then lysed in sodium dodecyl sulfate buffer containing protease inhibitors. DNA was sheared by sonication using a Sonic Dismembrator Model 500 (Fisher Scientific, Pittsburgh, PA, USA). Immunoprecipitation was carried out by incubation with 1 µg of anti-FLAG (M185-3L, MBL), anti-NANOG (A300-397A, Bethyl Laboratories) antibodies or rabbit IgG (Millipore) for 16 h. For qChIP assay, immunoprecipitated DNA was quantified by real-time qPCR using following primer sets; 5’-TGGCCAAAATATCCGAAAATTCCC-3’ (forward) and 5’-CAGTAGCTGGGACGAGCGTG-3’ (reverse). Each sample was assayed in triplicate, and the amount of precipitated DNA was calculated as the percentage of input sample.

### CTL-mediated apoptosis assay

CaSki and MDA-MB231 cells were labeled with CFSE (10 μM, Molecular Probes, Eugene, OR) in DMEM supplemented with 0.1% FBS. The CFSE-labeled CaSki cells were pulsed with MART1 peptide (10 μg mL^−1^) for 1 h. The CFSE-labeled MDA-MB231 cells and CaSki cells were mixed with cognate MART-1 or control noncognate NY-ESO1-specific CD8^+^ CTLs at a 1:1 ratio and incubated for 4 h at 37 °C. Cells were stained for active caspase-3 as an index of apoptosis and examined by flow cytometry as shown gating strategy in Supplementary Fig. 21.

### Granzyme B-mediated apoptosis assay

Recombinant human granzyme B (Enzo Life Sciences) was mixed with BioPorter Reagent (Sigma-Aldrich) at 25 °C for 5 min. Tumor cells were mixed with BioPorter–granzyme B complexes for 4 h at 37 °C. Cells were stained for active caspase-3 as an index of apoptosis and examined by flow cytometry.

### Trypan blue exclusion assay

For determining cell viability, trypan blue exclusion assay was performed. Briefly, cells were seeded at 1 × 10^5^ cells per well in 12-well plates 1 day prior to the assay. Chemical reagents were treated at the concentrations indicated in figures. After 24 h, cells were detached and stained with 0.4% trypan blue. Unstained cells were counted using a hemocytometer. Data are expressed as percentages of unstained cells compared with control cells not exposed to the chemical reagents.

### γ-irradiation-mediated viability assay

Cells were subjected to irradiation with a range of doses (0, 2, 4, 8, or 10 Gy) with a ^137^Cs radiator at a rate of 2.01 Gy/min and seeded into tissue culture plates. After culture for 96 h, viability was measured by trypan blue exclusion assay or active caspase-3 assay. After treatment of AUY922, cells were irradiated at 2 Gy.

### Tumor sphere-forming assay

Cells were plated at 1 × 10^3^ cells per well in six-well, super-low adherence vessels (Corning, Lowell, MA) containing serum-free DMEM-F12 (Thermo Scientific, Waltham, MA) supplemented with epidermal growth factor (20 ng mL^−1^), basic fibroblast growth factor (20 ng mL^−1^), and 1 × B27. Medium was replaced every 3 days to replenish nutrients. Colonies more than 50 μm in diameter were counted under a microscope.

### In vivo limiting dilution transplantation assay

To assess the role of HSP90A in tumor-initiating property of immune-edited CaSki P3 cells, we performed in vivo-limiting dilution transplantation assay using CaSki P3 cells stably transfected with empty vector (no insert), shHSP90AA1#1, or shHSP90AA1#2. Limiting doses of the tumor cells were subcutaneously injected into NOD/SCID mice. Six weeks after injection, mice were scored for the presence of palpable tumors. The frequency of tumorigenic cells and the 95% confidence interval were calculated using extreme limiting dilution analysis (ELDA)^59^.

### Luciferase assay

To determine the promoter activity of HSP90AA1, luciferase assay was performed. Briefly, the reporter construct, pGL3 basic, pGL3-HSP90AA1 WT, or pGL3-HSP90AA1 MUT together with pCMV-β-Gal, an internal control for transfection efficiency, were co-transfected into HEK293 cells using Lipofectamine 2000 (Invitrogen). After 24 h, cells were washed with PBS and lysed with Cell Culture Lysis Reagent (Promega). Luciferase activity was measured with a Turner Biosystems TD-20/20 luminometer after addition of 40 µl of luciferase assay reagent (Promega). Relative luciferase activity was normalized with the β-galactosidase activity in the cell lysate and calculated as an average of three independent experiments.

### Immunoprecipitation

HEK293 cells were transfected with pME18-myc/TCL1 for 24 h, and were lysed in NP40 lysis buffer (50 mM Tris–HCl, pH 8.0, 5 mM EDTA, 150 mM NaCl, 1% NP-40, 1 mM PMSF) containing protease inhibitor. Immunoprecipitation was carried out by incubation with 1 μg of anti-Myc antibody or mouse IgG for 16 h. The bound proteins were eluted by boiling in SDS sample buffer and were immunoblotted using anti-HSP90A antibody.

### In vivo ubiquitination assay

HA-tagged ubiquitin together with pME18-myc/TCL1 were co-transfected into HEK293 cells using Lipofectamine 2000 (Invitrogen). After 24 h, the cells were treated with 10 μM of MG132 for 8 h and lysed by incubation with two volumes of TBS containing 2% SDS at 95 °C for 10 min. After adding eight volumes of TBS containing 1% Triton X-100, lysates were sonicated and then immunoprecipitated with anti-Myc antibody coupled to TrueBlot anti-mouse Ig IP beads. The beads were washed with TBS, eluted by boiling in SDS sample buffer and immunoblotted using anti-HA antibody.

### Tissue samples and immunohistochemistry

Tissue microarrays (TMAs) containing four 1.0-mm-diameter cores retrieved from 483 formalin-fixed, paraffin-embedded tumor specimens, and matched nonadjacent normal specimens, were previously described^16^. The study subjects were compromised of 169 cervical cancers and 314 cervical intraepithelial neoplasia (CIN) patients who underwent surgical resection in Gangnam Severance Hospital between 1996 and 2010. Some of the paraffin blocks were provided by the Korea Gynecologic Cancer Bank through Bio & Medical Technology Development Program of the Ministry of Education, Science and Technology, Korea (NRF-2017M3A9B8069610). Tissue samples were collected from patients who had signed informed consent form. This study was approved by the Institutional Review Board of Gangnam Severance Hospital (Seoul, South Korea), and all procedures were conducted in accordance with the guidelines of the Declaration of Helsinki.

The TMA sections were deparaffinized by xylene and then rehydrated through a graded ethanol series. Antigen retrieval was performed in heat-activated antigen retrieval pH 6.0 (Dako, Carpinteria, CA). Endogenous peroxidase activity was quenched with 3% H_2_O_2_ for 10 min. The sections were incubated for 30 min with mouse monoclonal anti-HSP90A antibodies (Abcamz, Cambridge, MA; Clone 4F10) at 1:4000 dilutions, followed by a standard ABC protocol using EnVision^+^ Dual Link System-HRP (Dako). The reactions were visualized using 3,3-diaminobenzadine substrate for 10 min and lightly counter-stained with hematoxylin. Negative controls including immunoglobulin G (IgG) and omission of the primary antibody were concurrently performed, and the TMA included appropriate positive control tissues. NANOG protein expression was previously evaluated in the same cohort^16^.

For digital image analysis, all stained slides were scanned using the NanoZoomer 2.0 HT (Hamamatsu Photonics K.K., Japan) at ×40 objective magnification (0.23 μm per pixel resolution). The images were analyzed using Visiopharm software v6.2.0.2089 (Visiopharm, Hørsholm, Denmark). Tumor nuclei were defined after training the system. Cytoplasm was further defined by outlining the defined nucleus. DAB intensity was obtained by using a predefined algorithm ranging from 0 (no signal) to 300 (fully saturated signal), as described previously^60^.

### Bioinformatic analyses from published clinical database

RNA-seq datasets from three cancer types (cholangiocarcinoma, testicular germ cell tumors, and uveal melanoma) at the Cancer Genome Atlas (TCGA) portal (https://tcga-data.nci.nih.gov/tcga/)^61^ were used to calculate a correlation coefficient (*r*) between NANOG and HSP90AA1 expressions. To investigate the clinical relevance in patients treated with anti-PD-1 therapy, we used the published datasets (GSE91061)^29^. RNA-seq read counts were log_2_ normalized, and unbiased median gene expression value cutoffs were applied for the analysis of high/low expression subgroups and their potential associations with overall disease outcome (days to death or last follow-up).

To explore the gene sets transcriptionally regulated by NANOG and IRF8, we analyzed DEGs using TF-LOF Expression from gene expression omnibus (GEO)^62^. Briefly, DEGs were then identified for NR versus R to anti-PD-1 therapy at thresholds of FDR < 0.05, 1.5FC. The unbiased lists of significantly up-regulated and down-regulated genes were used to conduct upstream regulatory analysis using TF-LOF expression analysis. A preliminary NANOG-responsive genes was derived using the TF analysis and validated followed by filtering to overlap genes that have been previously identified as responding to NANOG^34–38^. The expression of multi-genes was calculated by averaging of the included genes for the NANOG-responsive genes (six-gene: FGFBP1, SPESP1, HMGA2, PERP, FGF1, and DKK1) and IRF8-responsive genes (24-gene: CD74, CIITA, LY86, ZBTB32, FLT3LG, DAPP1, RNASE4, TAPBPL, LSP1, CTSS, SOCS1, HLA-DMB, ELF4, KYNU, UCP2, TLR9, BLNK, HLA-DRA, LPXN, IRF5, CD37, SLC15A3, MS4A1 and GPR18) as described previously^63^.

### Tumor treatment experiments

NOD/SCID mice were inoculated subcutaneously with 2 × 10^6^ MDA-MB231 P3 cells per mouse. Seven days following tumor challenge, AUY922 (0.05 mg kg^−1^) or PBS was treated via intraperitoneal route. Following day after AUY922 treatment, mice received adoptive transfer with 2 × 10^6^ MART-1-specific CTLs. This treatment regimen was repeated for three cycles. Mice were monitored for tumor burden and survival for 21 and 40 days after challenge, respectively. C57BL/6 mice were inoculated subcutaneously with 1 × 10^6^ tumor cells per mouse. Between 5 and 13 days following tumor challenge, AUY922 (0.05 mg kg^−1^) or PBS was treated via intraperitoneal route. Following day after AUY922 treatment, mice administered anti-PD-1 (BioXcell, NH, USA) or isotype antibody control that was administrated every 3 days at a dose of 200 μg per mice in accordance with the schedule described in Fig. 8a and Supplementary Fig. 20a. This treatment regimen was repeated for three cycles. Mice were monitored for tumor burden and survival for 20 and 40 days after challenge, respectively.

### Immune cell tumor infiltration

Treated C57BL/6 mice were sacrificed on day 15 following tumor inoculation and tumors were harvested. Tumors were dissected into fragments by cutting, dissociated by a cell strainer. Cell suspensions were stained for intracellular and extracellular protein markers of interest. Stained samples were assessed on a flow cytometer (BD biosciences, San, USA) along with CellQuest Pro software. Staining antibodies were as follows: anti-CD3 (BD biosciences, San, USA), anti-CD8 (BD biosciences, San, USA), anti-granzyme B (BD biosciences, San, USA), and anti-IFN-γ (BD biosciences, San, USA).

### Analysis of generation of antigen-specific T cells

To investigate the generation of tumor antigen-specific CD8^+^ T cells, treated C57BL/6 mice were sacrificed on day 15 following tumor inoculation and tumors, drain lymph nodes and spleens were harvested and incubated with either DMSO or gp100 peptide (KVPRNQDWL) at 1 μg mL^−1^ for 24 h and stained with anti-CD8. Cell fixation and permeabilization were conducted thereafter with the CytoFix/Cytoperm^TM^ kit (BD biosciences, San, USA), followed by washing with cold PBS and labeling with anti-IFN-γ. The flow cytometer along with CellQuest Pro software was used to quantify the number of antigen-specific IFN-γ^+^, CD8^+^ T cells.

### Statistical analysis

All data are representative of at least three separate experiments. Statistical differences were calculated by either Student’s *t*-test (two-tailed, unpaired), one-way ANOVA, or two-way ANOVA using GraphPad Prism software. For tissue samples and immunohistochemistry, statistical analyses were performed using IBM SPSS statistics version 21 (IBM Corporation, Armonk, NY). The statistical significance of the differences in the protein expressions in the different groups was calculated by the Mann–Whitney *U* test. Chi-square test was used to assess associations between protein expressions and tumor size. Spearman’s rank correlation analysis was used to evaluate the association between protein markers. Survival curves were calculated using the Kaplan–Meier method and the difference between the survival curves was calculated by the log-rank test. A Cox proportional hazards model was created to identify independent predictors of survival. Results with two-tailed *p*-values < 0.05 were considered statistically significant.

### Reporting summary

Further information on research design is available in the Nature Research Reporting Summary linked to this article.

## Supplementary information

Supplementary Information

Reporting Summary

## Data Availability

RNA sequencing data have been deposited in the Gene Expression Omnibus (GEO) under the accession code GSE91061 and the Cancer Genome Atlas (TCGA) portal [https://tcga-data.nci.nih.gov/tcga]. The TF analysis and ELDA that support the findings of this study are publicly available online at [https://amp.pharm.mssm.edu/Enrichr/] and [http://bioinf.wehi.edu.au/software/elda/], respectively. Figures 1b–g, 2b, d, f, h, j, 3b, d–g, 4a, b, h, 5a–e, 6b–d, f–h, 7a–g, and 8b–h, and Supplementary Figs. 2, 3b, 4b, 5a, c–e, 6a–d, 7, 8a, b, 9a, b, 10a, b, 11a–c, 13b,c, 14a,b, 15a, c, 16a–e, 17a,b, 18d, 19, and 20b–d are provided in the Source Data file. The raw images for the immunoblots are provided in the supplementary information. All other data supporting the findings of this study are available from the corresponding author on reasonable request.

## Source data

Source Data
