# Supplementary material for: HSP90A inhibition promotes anti-tumor immunity by reversing multi-modal resistance and stem-like property of immune-refractory tumors
Source: Nat Commun. 2020 Jan 28;11:562. doi: 10.1038/s41467-019-14259-y (PMC6987099; doi:10.1038/s41467-019-14259-y)
Supplement: Supplementary file 1 — Supplementary Information [file 41467_2019_14259_MOESM1_ESM.pdf]

Supplementary information for the manuscript entitled:

HSP90A inhibition promotes anti-tumor immunity by reversing multi-modal resistance and stem-like property of immune-refractory tumors

Kwon-Ho Song et al.

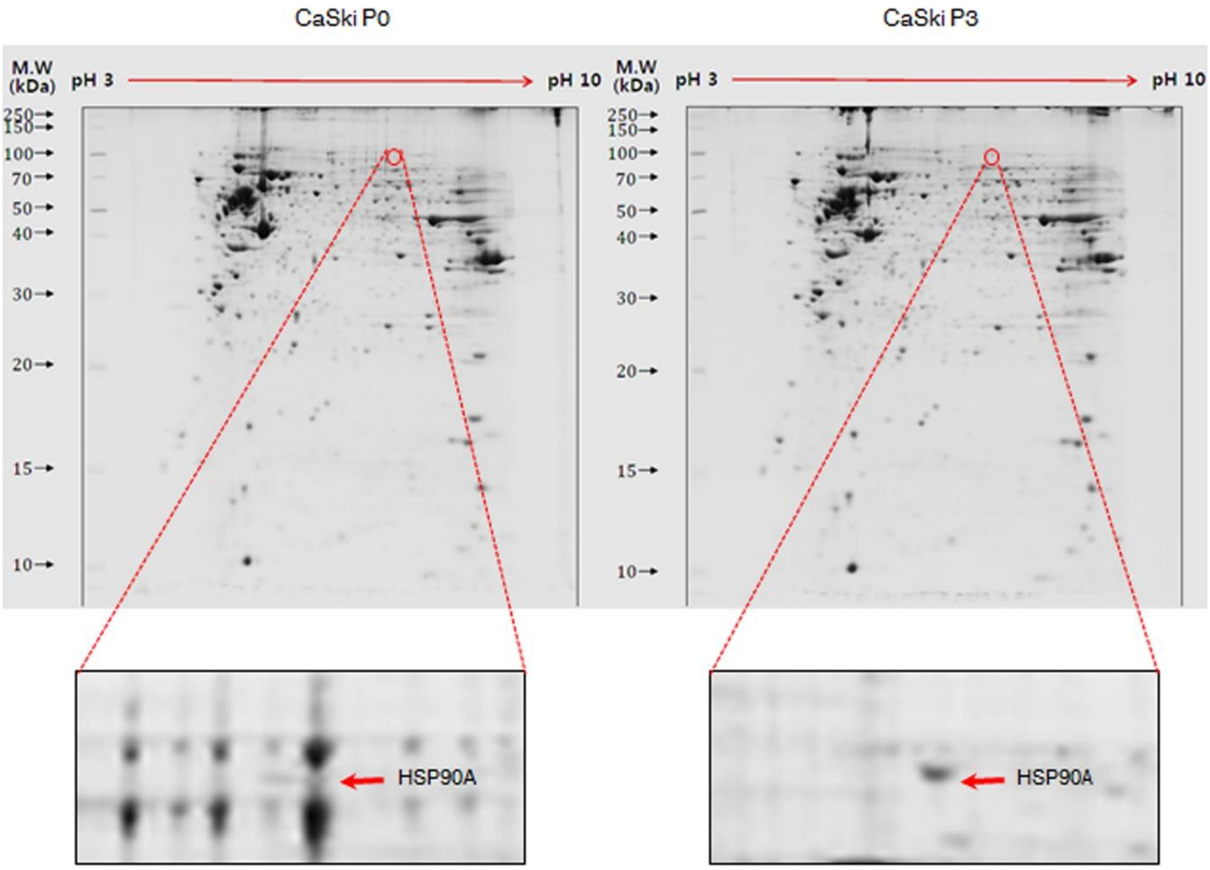

Supplementary Fig. 1 Identification of HSP90A as an upregulated protein in tumor cells after immune editing. 2-D Coomassie Brilliant Blue G-250 gel electrophoresis of total proteins from CaSki P0 (left) or P3 (right). Among proteins upregulated in P3 cells relative to P0 cells, HSP90A was identified by mass spectrometry analysis. This experiment was performed in triplicate.

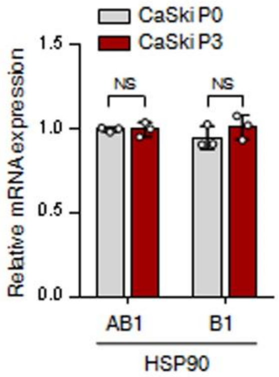

Supplementary Fig. 2 Expression levels of HSP90AB1 and HSP90B1 in CaSki P0 and P3 cells. mRNA expression of HSP90AB1 encoding HSP90B and HSP90B1 encoding GRP-94 were determined by qRT-PCR. Graphs represent three independent experiments performed in triplicate. The *p*-value is calculated by 2-tailed Student's t test. NS, not significant. Error bars represent standard deviations from the mean.

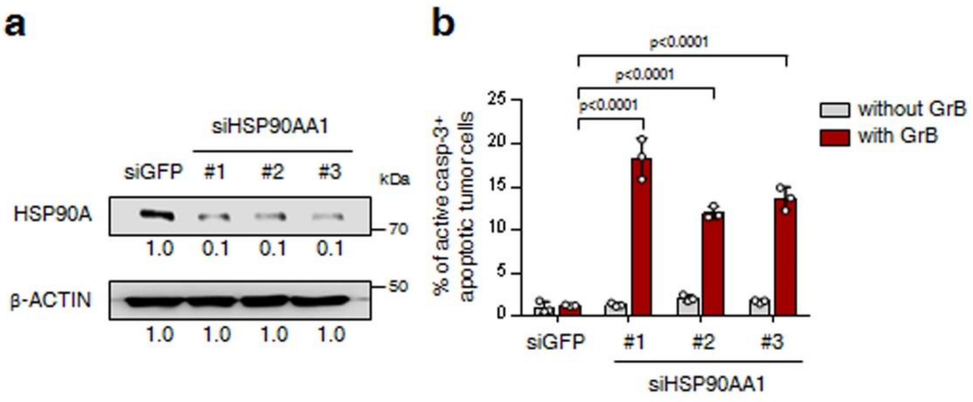

Supplementary Fig. 3 Silencing of HSP90AA1 reverses immune-resistant phenotype of CaSki P3 cells. CaSki P3 cells were transfected with siGFP, siHSP90AA1-#1, -#2, or -#3. **a** Level of HSP90A in these cells was probed by Western blot. This experiment was performed in triplicate. **b** Flow cytometry analysis of the active caspase-3$^+$ cells in the cells after intracellular delivery of granzyme B. Graphs represent three independent experiments performed in triplicate (N=3). The *p*-values by two-way ANOVA are indicated. Data represent the mean ± SD.

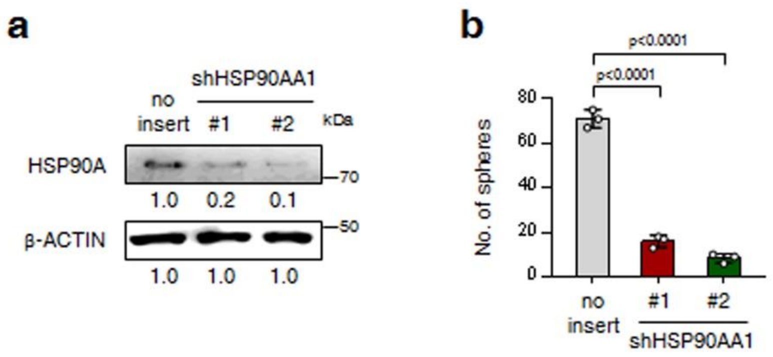

Supplementary Fig. 4 Stable knockdown of HSP90AA1 reduces CSC-like property of CaSki P3 cells. CaSki P3 cells were stably transfected with empty vector (no insert), shHSP90AA1#1, or shHSP90AA1#2. **a** Level of HSP90A in these cells was probed by Western blot. **b** Compared to P3-no insert cells, P3-shHSP90AA1#1 and P3-shHSP90AA1#2 cells had reduced sphere-forming capacity in low-density suspension culture. All experiments were performed in triplicate. The *p*-values by one-way ANOVA are indicated **(b)**. Data represent the mean ± SD.

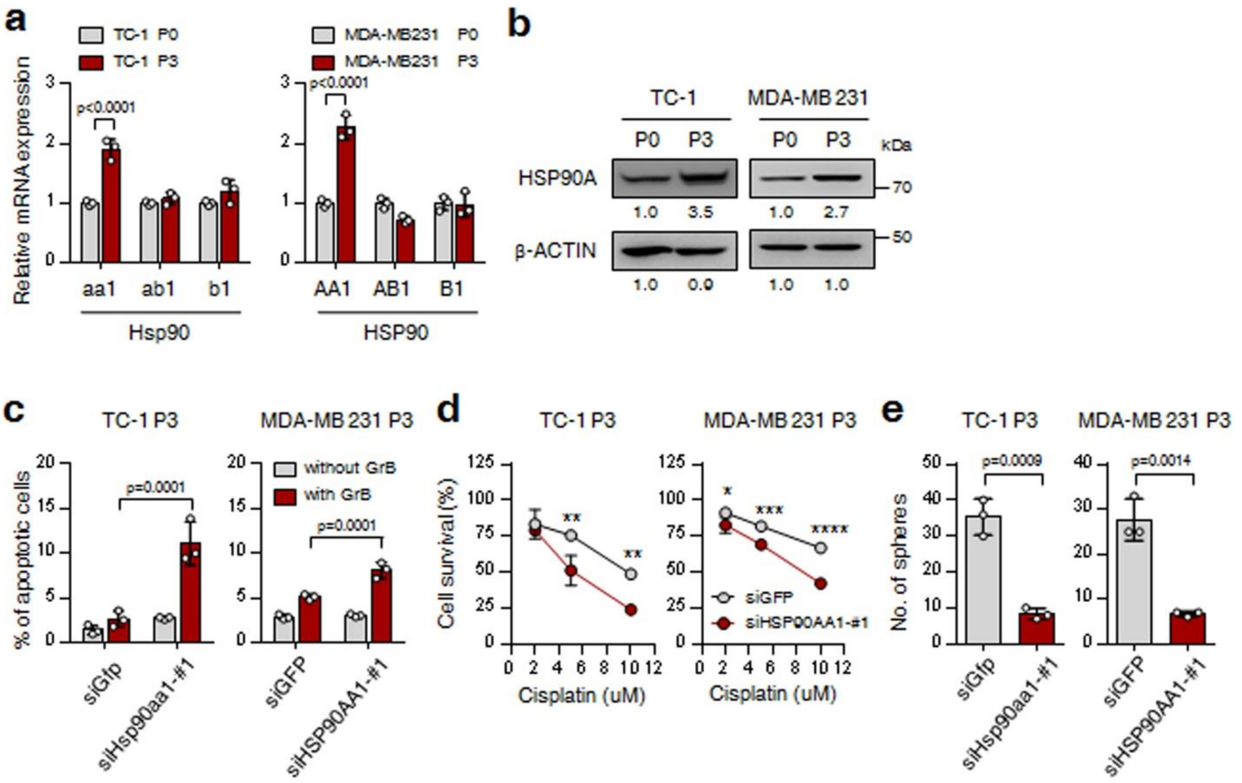

Supplementary Fig. 5 HSP90A is crucial for multi-aggressive properties of immune-edited tumor cells. **a** mRNA levels of Hsp90aa1, Hsp90ab1, and Hsp90b1 (TC-1), or HSP90AA1, HSP90AB1, and HSP90B1 (MDA-MB231) in TC-1 or MDA-MB231 P0 and P3 cells were determined by qRT-PCR. **b** HSP90A protein level in TC-1 or MDA-MB231 P0 and P3 cells were determined by Western blot. β-ACTIN was included as an internal loading control. Numbers below blot images indicate the expression as measured by fold change. **c-e** TC-1 P3 or MDA-MB231 P3 cells were transfected with the indicated siRNAs. **c** The frequency of apoptotic (active caspase-3^+) cells after intracellular delivery with or without granzyme B (GrB) was estimated by flow cytometry analysis. **d** Cells were treated with indicated concentrations of cisplatin. The percentage of viable cells was determined by trypan blue exclusion assay at 24h after cisplatin treatment. (**p* < 0.05, ***p* < 0.01, ***p* < 0.001 and ****p* < 0.0001) **e** Sphere-forming capacity of the cells in low-density suspension culture. All experiments were performed in triplicate. The *p*-values by 2-tailed Student's t test **(a and e)** or two-way ANOVA **(c and d)** are indicated. Error bars represent standard deviations from the mean.

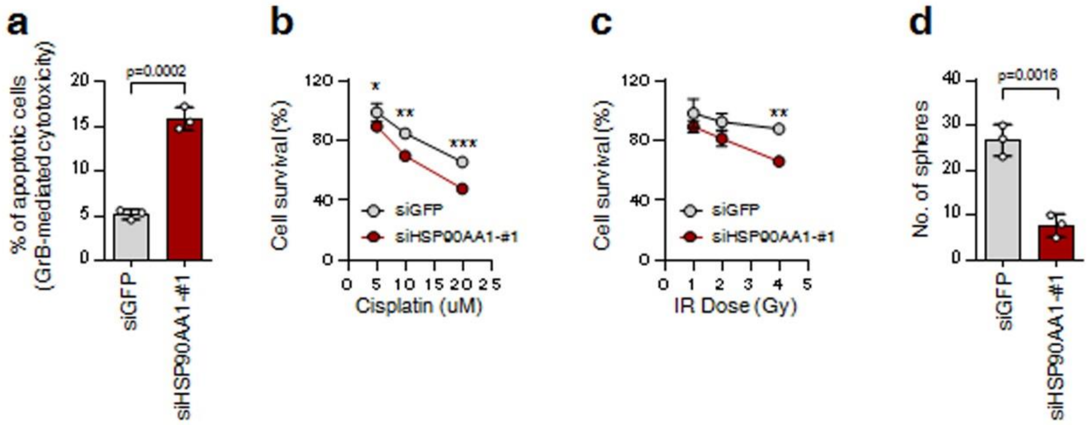

Supplementary Fig. 6 HSP90AA1 expression is crucial for NANOG-mediated aggressive phenotypes. CaSki-NANOG cells were transfected with siGFP or siHSP90AA1-#1. **a** Flow cytometry analysis of the frequency of apoptotic (active caspase-3^{+}) cells in the cells after intracellular delivery of granzyme B. **b and c** Cells were treated with indicated concentrations of cisplatin **(b)** and irradiation **(c)**. The percentage of viable cells was determined by trypan blue exclusion assay at 24h after either cisplatin treatment or irradiation. (*$p < 0.01$, **$p < 0.001$ and ***$p < 0.0001$) **d** Sphere-forming capacity of the cells in low-density suspension culture. All experiments were performed in triplicate. The *p*-values by 2-tailed Student's t test **(a and d)** or two-way ANOVA **(b and c)** are indicated. Error bars represent standard deviations from the mean.

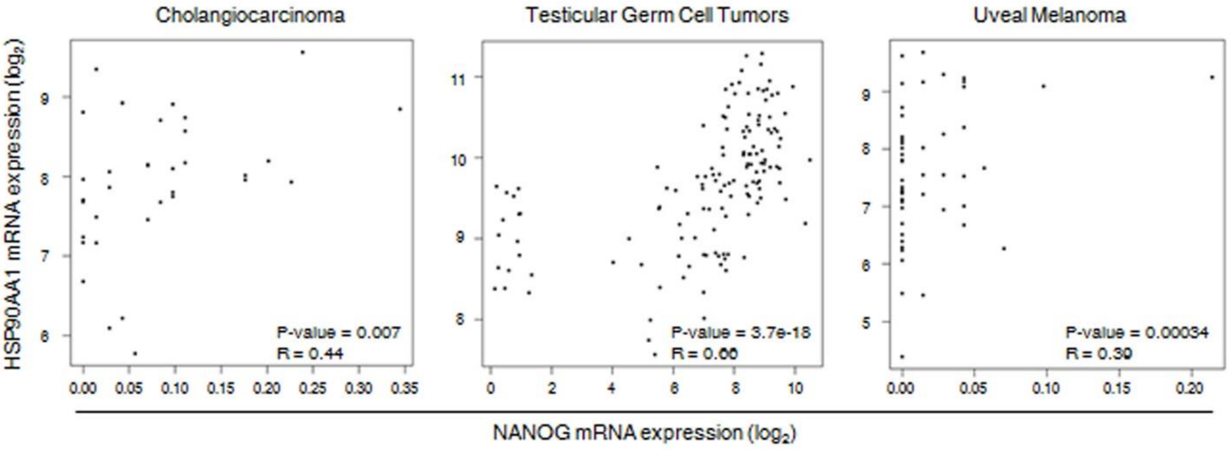

Supplementary Fig. 7 Correlation analysis between NANOG and HSP90AA1 in multiple human cancer. RNA-seq datasets from 3 cancer types, Cholangiocarcinoma (left, N=36, Spearman R = 0.44, p = 0.007), Testicular Germ Cell Tumors (middle, N=137, Spearman R = 0.66, p = 3.7 x 10^{-18}) and Uveal Melanoma (right, N=79, Spearman R = 0.39, p = 3.4 x 10^{-4}) at the Cancer Genome Atlas (TCGA) portal were used to calculate a correlation coefficient (r) between NANOG and HSP90AA1 expression. Correlation analysis was performed using Spearman correlation coefficient (R).

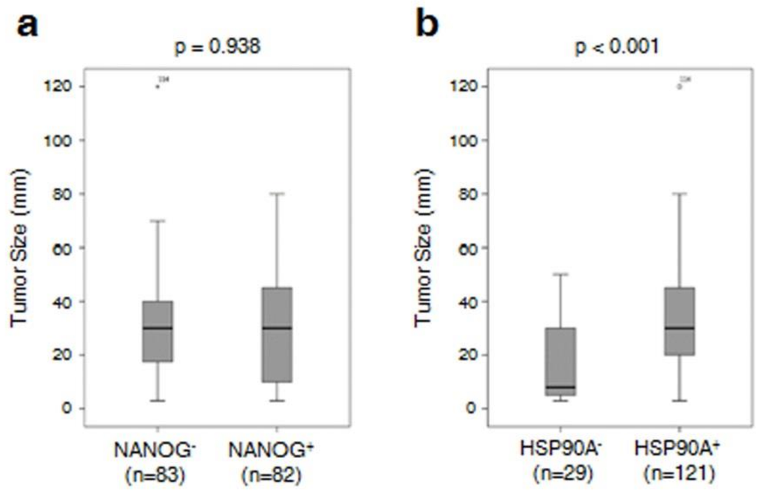

Supplementary Fig. 8 Association of NANOG or HSP90A, and tumor size in cervical cancer patients. **a** High level of NANOG showed tendency of large-sized tumor. **b** High expression of HSP90A ($p < 0.001$) was strongly associated with large-sized tumor. The *p*-value by Mann–Whitney U test **(a and b)** is indicated. Error bars represent standard deviations from the mean. In the box plots, the top and bottom edges of boxes indicate the first and third quartiles, respectively; the center lines indicate the medians; and the ends of whiskers indicate the maximum and minimum values, respectively.

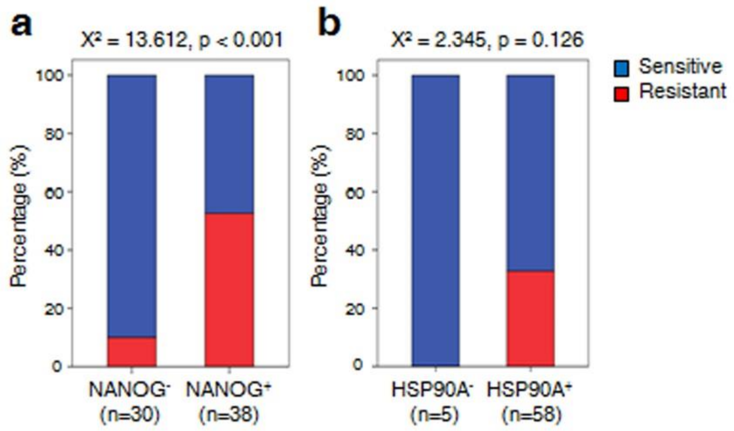

Supplementary Fig. 9 Association between NANOG or HSP90A level, and chemoradiation sensitivity in patients with cervical cancer. **a** High level of NANOG (p < 0.001) was significantly associated with chemoradiation resistance. **b** High level of HSP90A exhibited tendency of chemoradiation resistance. p-values were determined by Mann–Whitney U test **(a and b).**

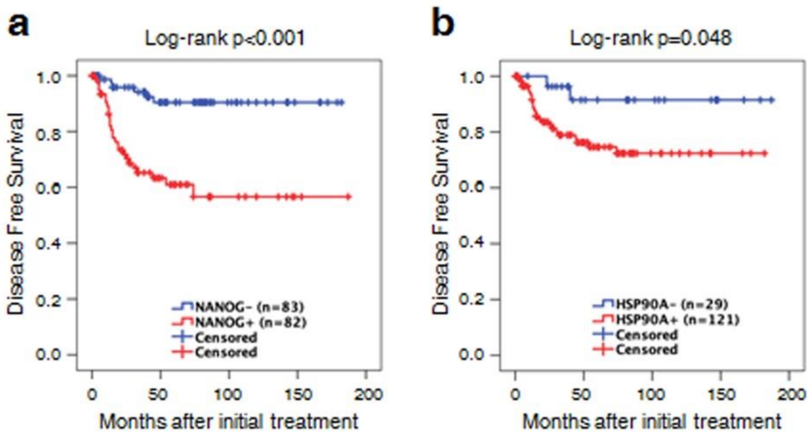

Supplementary Fig. 10 Kaplan-Meier plots of disease-free survival for cervical cancer patients according to NANOG or HSP90A level. **a** High level of NANOG was significantly associated with short disease-free survival (p < 0.001). **b** High level of HSP90A was associated with poor disease-free survival (p = 0.048). p-values by Log-rank (Mantel-Cox) test are indicated **(a and b).**

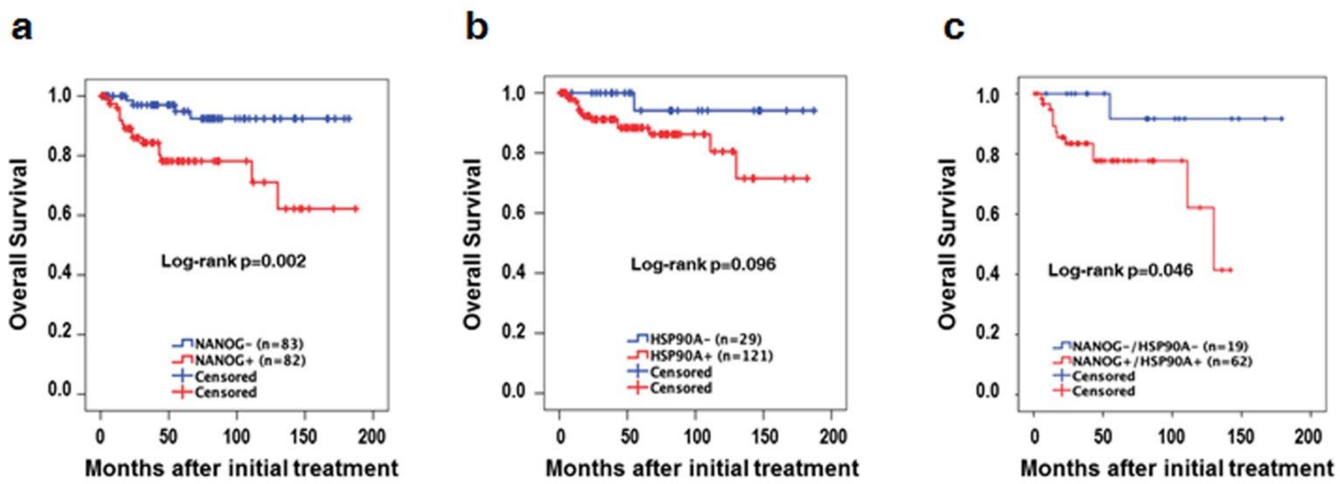

Supplementary Fig. 11 Kaplan-Meier plots of overall survival for cervical cancer patients according to NANOG and HSP90A level. **a** High level of NANOG significantly displayed short overall survival (*p* = 0.002). **b** High level of HSP90A exhibited tendency of short overall survival. **c** Patients with NANOG^{+}/HSP90A^{+} level displayed worse overall survival (p = 0.046) than patients with other groups. *p*-values were determined by Log-rank (Mantel-Cox) test **(a-c)**

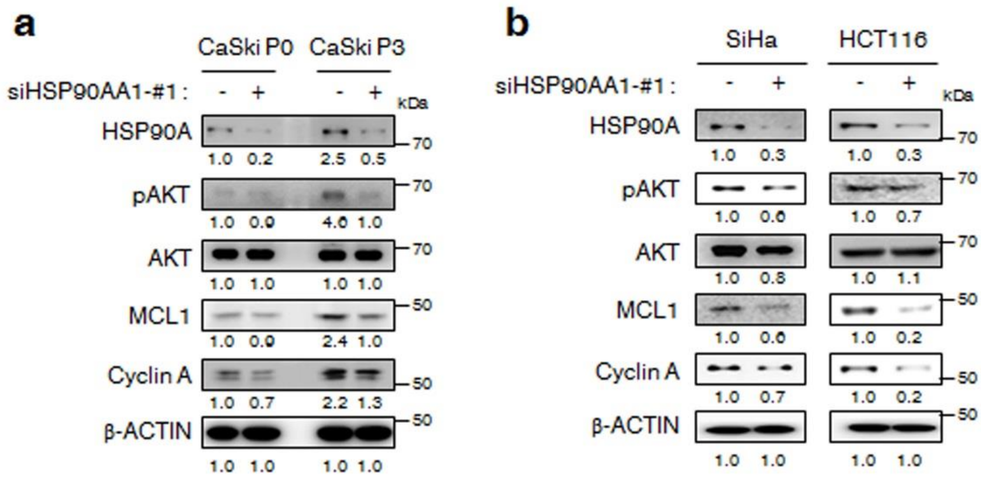

Supplementary Fig. 12 NANOG-HSP90A axis is conserved in multiple types of human cancer cells. **a** CaSki P0 and P3 cells were transfected siGFP or siHSP90AA1-#1. **b** SiHa and HCT116 were transfected siGFP or siHSP90AA1-#1. Levels of HSP90A, pAKT, AKT, MCL1 and Cyclin A were proved by Western blot. β-ACTIN was included as an internal loading control. Numbers below blot images indicate the expression as measured by fold change. All experiments were performed in triplicate.

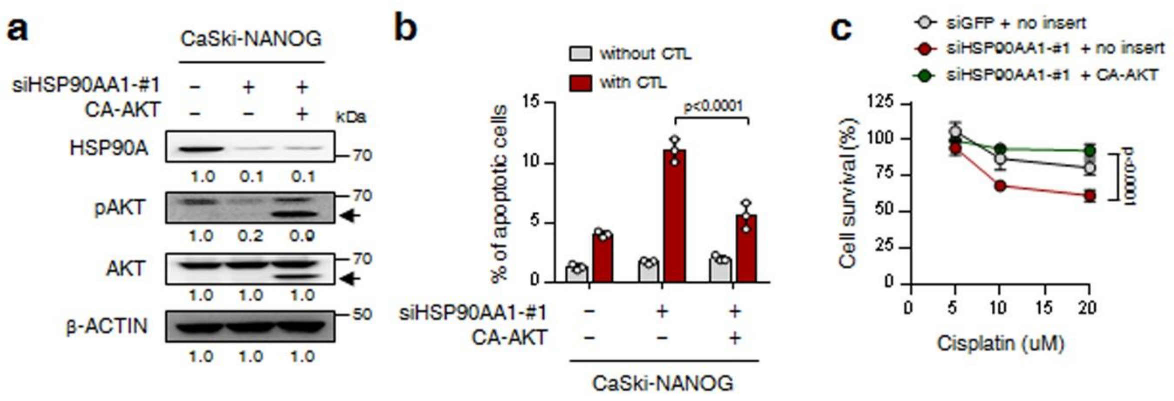

Supplementary Fig. 13 Overexpression of constitutively active AKT (CA-AKT) restores the susceptibility of HSP90AA1-depleted tumor cells to immunotherapy and chemotherapy. **a-c** CaSki NANOG cells were transfected with siGFP or siHSP90AA1-#1, and then transfected with CA-AKT constructs at 24h after siRNA transfection. **a** Level of HSP90A, pAKT, and AKT in these cells was probed by western blot. CA-AKT displayed greater mobility than endogenous AKT due to deletion of its negative-regulatory domain, as indicated by arrows. β-ACTIN was included as an internal loading control. Numbers below blot images indicate the expression as measured by fold change. **b** The frequency of apoptotic (active caspase-3^{+}) cells in the MART-1 peptide pulsed cells after incubation with or without MART-1 specific CTLs at a 1:1 ratio for 4 h was estimated by flow cytometry analysis. **c** Cells were treated with indicated concentrations of cisplatin. The percentage of viable cells was determined by trypan blue exclusion assay at 24 h after cisplatin treatment. Graphs represent three independent experiments performed in triplicate (N=3). All experiments were performed in triplicate. The *p*-values by two-way ANOVA are indicated **(b and c)**. Data represent the mean ± SD.

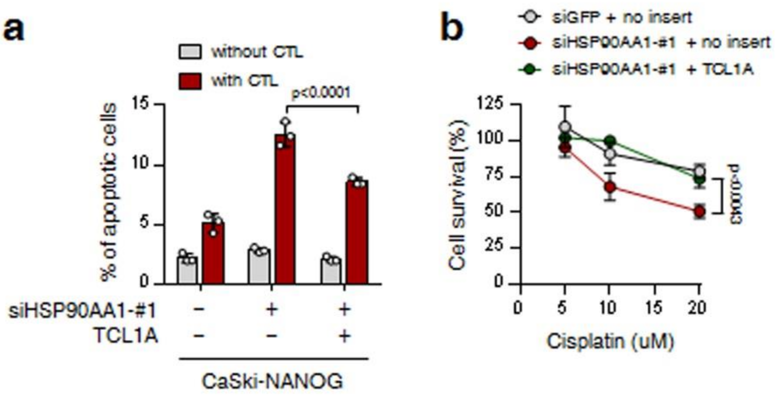

Supplementary Fig. 14 Restoration of TCL1A reverses the susceptibility of HSP90AA1-depleted tumor cells to immunotherapy and chemotherapy. CaSki NANOG cells were transfected with siGFP or siHSP90AA1-#1, and then transfected with TCL1A constructs at 24h after siRNA transfection. **a** The frequency of apoptotic (active caspase-3^{+}) cells in the MART-1 peptide pulsed cells after incubation with or without MART-1 specific CTLs at a 1:1 ratio for 4 h was estimated by flow cytometry analysis. **b** Cells were treated with indicated concentrations of cisplatin. The percentage of viable cells was determined by trypan blue exclusion assay at 24 h after cisplatin treatment. Graphs represent three independent experiments performed in triplicate (N=3). The *p*-values by two-way ANOVA are indicated **(a and b)**. Data represent the mean ± SD.

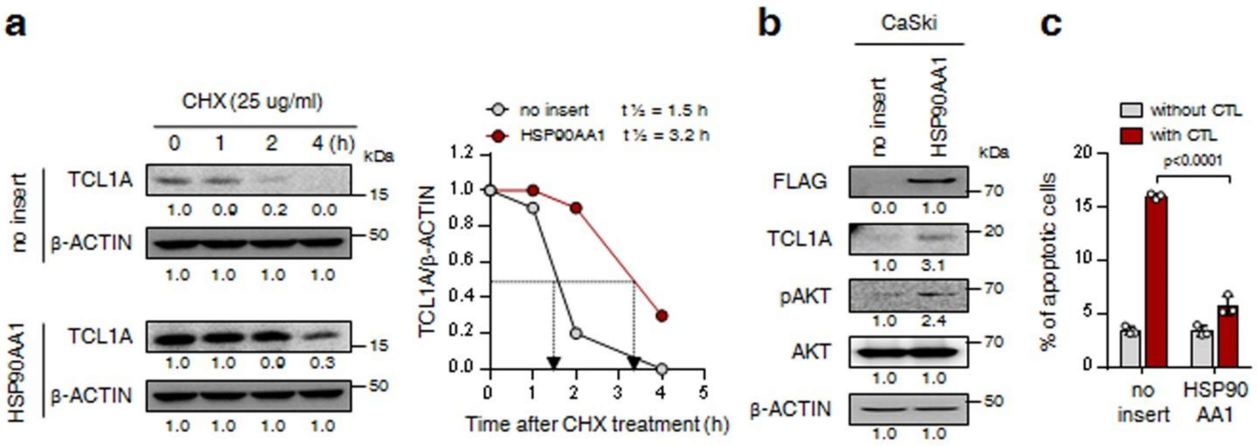

Supplementary Fig. 15 HSP90AA1 overexpression induces AKT activation through TCL1A stabilization and promotes resistance to CTLs. **a-c** CaSki P0 cells were transfected with empty vector (no insert) or HSP90AA1-FLAG constructs. **a** At 24 h after HSP90AA1-FLAG transfection, the cells were treated with cycloheximide (25 ug mL^{-1}, CHX) for the indicated times. Cell lysates were subjected to immunoblotting with anti-TCL1A antibodies. Graph represents the means ± SD of three quantified data, after normalization to the corresponding β-ACTIN level. **b** Levels of FLAG (HSP90A), TCL1A, pAKT, and AKT were analyzed by western blotting. β-ACTIN was included as an internal loading control. Numbers below blot images indicate the expression as measured by fold change (**a and b**) These experiments were performed in triplicate. **c** The frequency of apoptotic (active caspase-3^+) cells in the MART-1 peptide pulsed cells after incubation with or without MART-1 specific CTLs at a 1:1 ratio for 4 h was estimated by flow cytometry analysis. Graphs represent three independent experiments performed in triplicate (N=3). The *p*-value by two-way ANOVA is indicated. Data represent the mean ± SD.

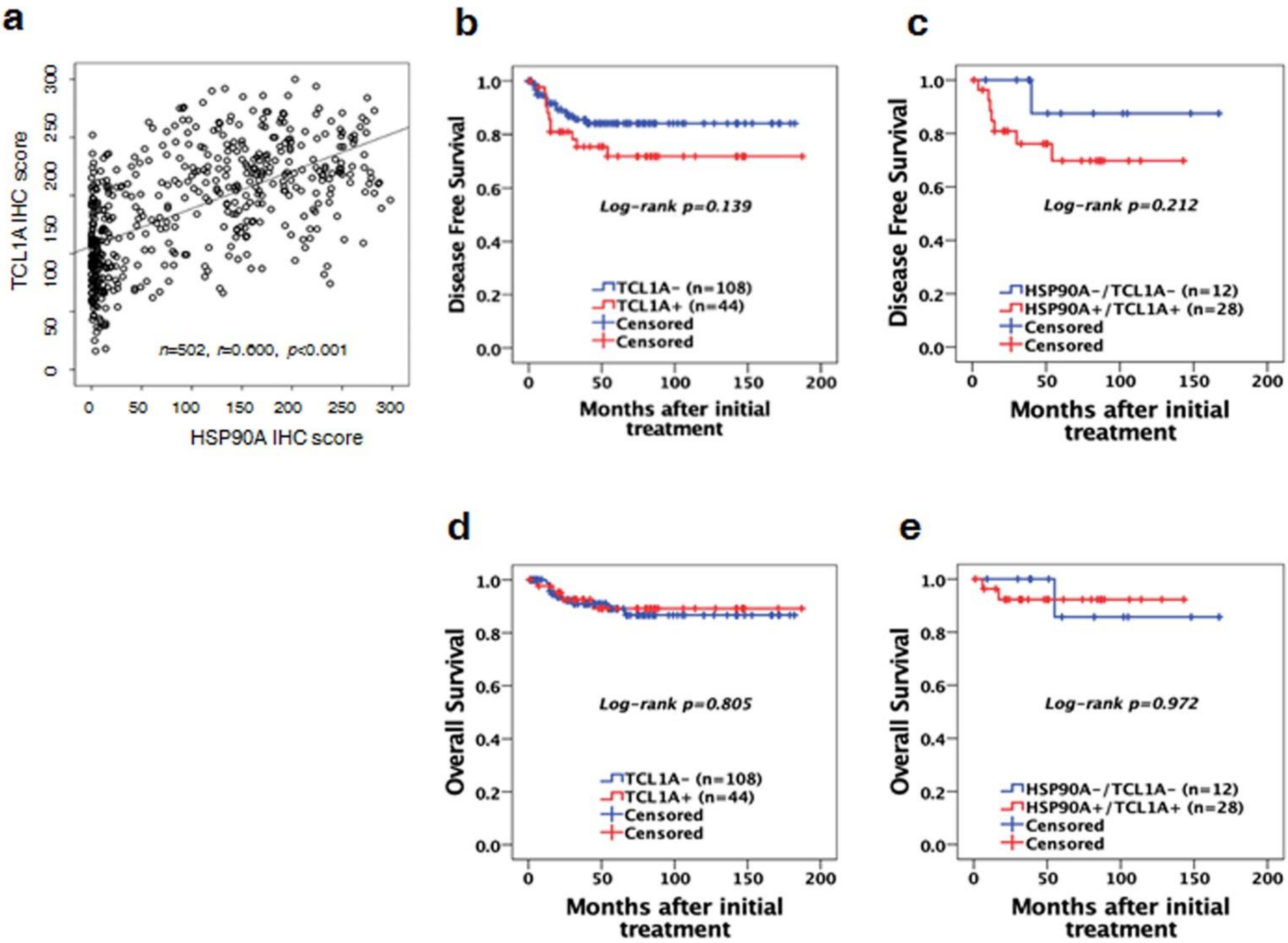

Supplementary Fig. 16 Correlation between HSP90A and TCL1A level in cervical cancer patients, and the relationship of TCL1A or combined HSP90A^{+}/TCL1A^{+} level with patient's survival outcomes. **a** Correlation between NANOG and HSP90A level in patients with cervical cancer (Spearman's $r = 0.600$, $p < 0.001$). **b** Patients with TCL1A^{+} level showed tendency of worse disease-free survival than patients with TCL1A^{-} level. **c** HSP90A^{+}/TCL1A^{+} patients exhibited tendency of worse disease-free survival than HSP90A^{-}/TCL1A^{-} patients. TCL1A^{+} **(d)** and HSP90A^{+}/TCL1A^{+} **(e)** level in patients was not associated with overall survival in human cervical cancer. *p*-values were determined by Log-rank (Mantel-Cox) test **(b-e).**

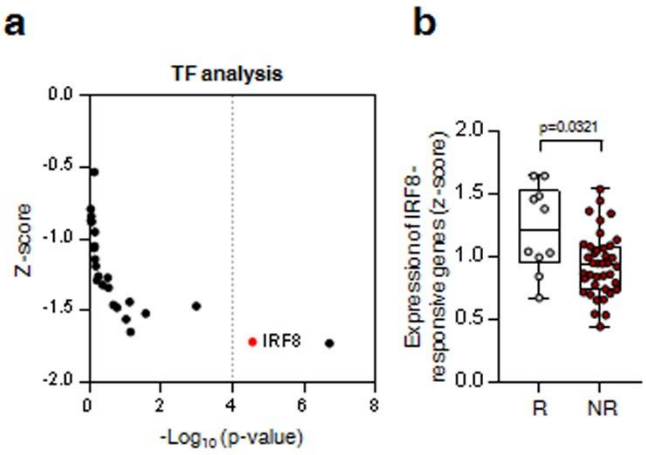

Supplementary Fig. 17 IRF8-responsive genes were decreased in non-responder to PD-1 blockade therapy. **a** TF analysis of down-regulated DEGs in non-responders relative to responders to anti-PD-1 therapy. Each dot represents one TF which has transcription factor motifs enriched in DEGs (IRF8, p = 2.74 x 10^{-5}). The broken line indicates p-value cutoff (p=0.0001). The p-value is calculated by Fisher exact test. **b** Comparisons of expression level of IRF8-responsive genes in the responder (R, N=10) and non-responder (NR, N=39). The top and bottom edges of boxes indicate the first and third quartiles, respectively; the center lines indicate the medians, and the ends of whiskers indicate the maximum and minimum values, respectively. Error bars represent standard deviations from the mean. The p-value by unpaired t-test with Welch's correction is indicated.

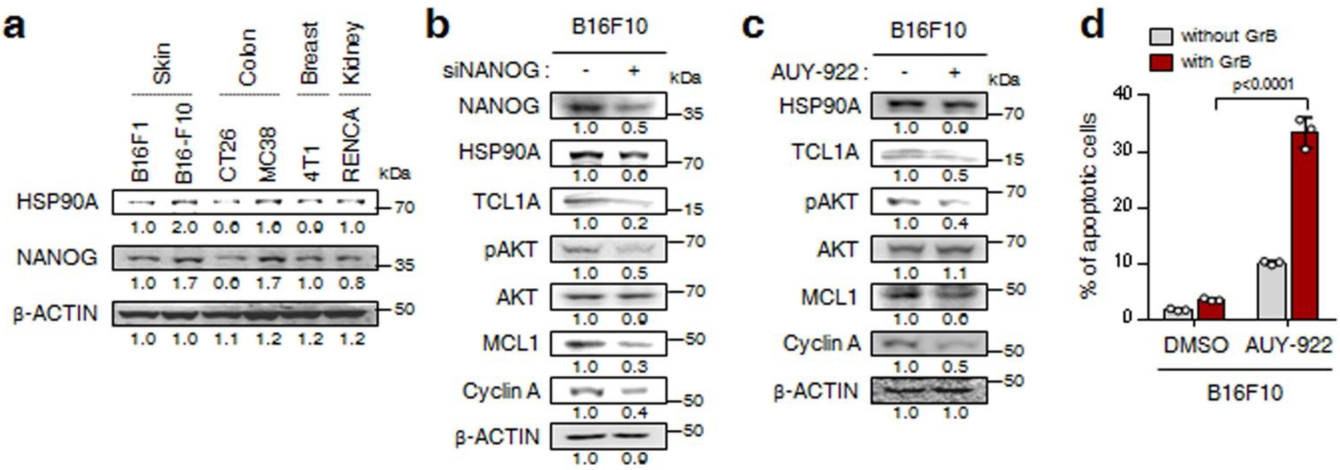

Supplementary Fig. 18 NANOG-HSP90A axis are conserved in the immune-refractory B16F10 tumor cells. **a** Western blot analysis of HSP90A and NANOG expression in various mouse cancer cell lines. **b** B16F10 cells were transfected with siGFP or siNANOG. Western blot analysis of the expression of NANOG, HSP90A, TCL1A, pAKT, AKT, MCL1, and Cyclin A. **c and d** B16F10 cells were treated with DMSO or AUY-922. **c** Western blot analysis of HSP90A, TCL1A, pAKT, AKT, MCL1, and Cyclin A. **d** Flow cytometry analysis of apoptotic tumor (active caspase-3^{+}) cells in the cells after intracellular delivery of granzyme B. β-ACTIN was included as an internal loading control. Numbers below blot images indicate the expression as measured by fold change (**a-c**). All experiments were performed in triplicate. The *p*-value by two-way ANOVA is indicated. Data represent the mean ± SD.

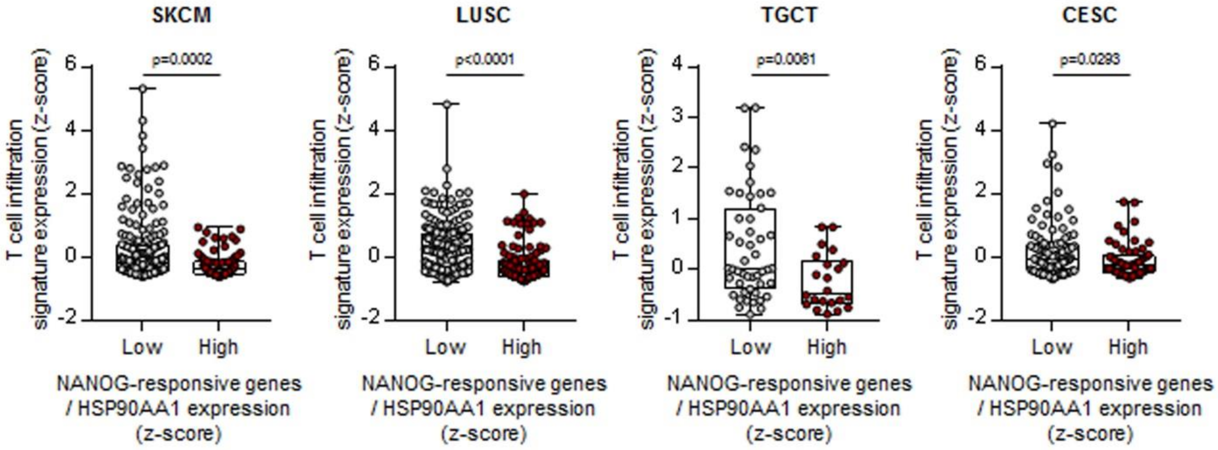

Supplementary Fig. 19 Comparisons of T cell infiltration gene signature in TCGA cohort with low levels and high levels of both NANOG-responsive genes and HSP90AA1. Error bars represent standard deviations from the mean. The *p*-values by unpaired t-test are indicated. (SKCM; N=274, LUSC; N=340, TGCT; N=70 or CESC; N=160 biologically independent tumor samples from TCGA cohort). The top and bottom edges of boxes indicate the first and third quartiles, respectively; the center lines indicate the medians, and the ends of whiskers indicate the maximum and minimum values, respectively.

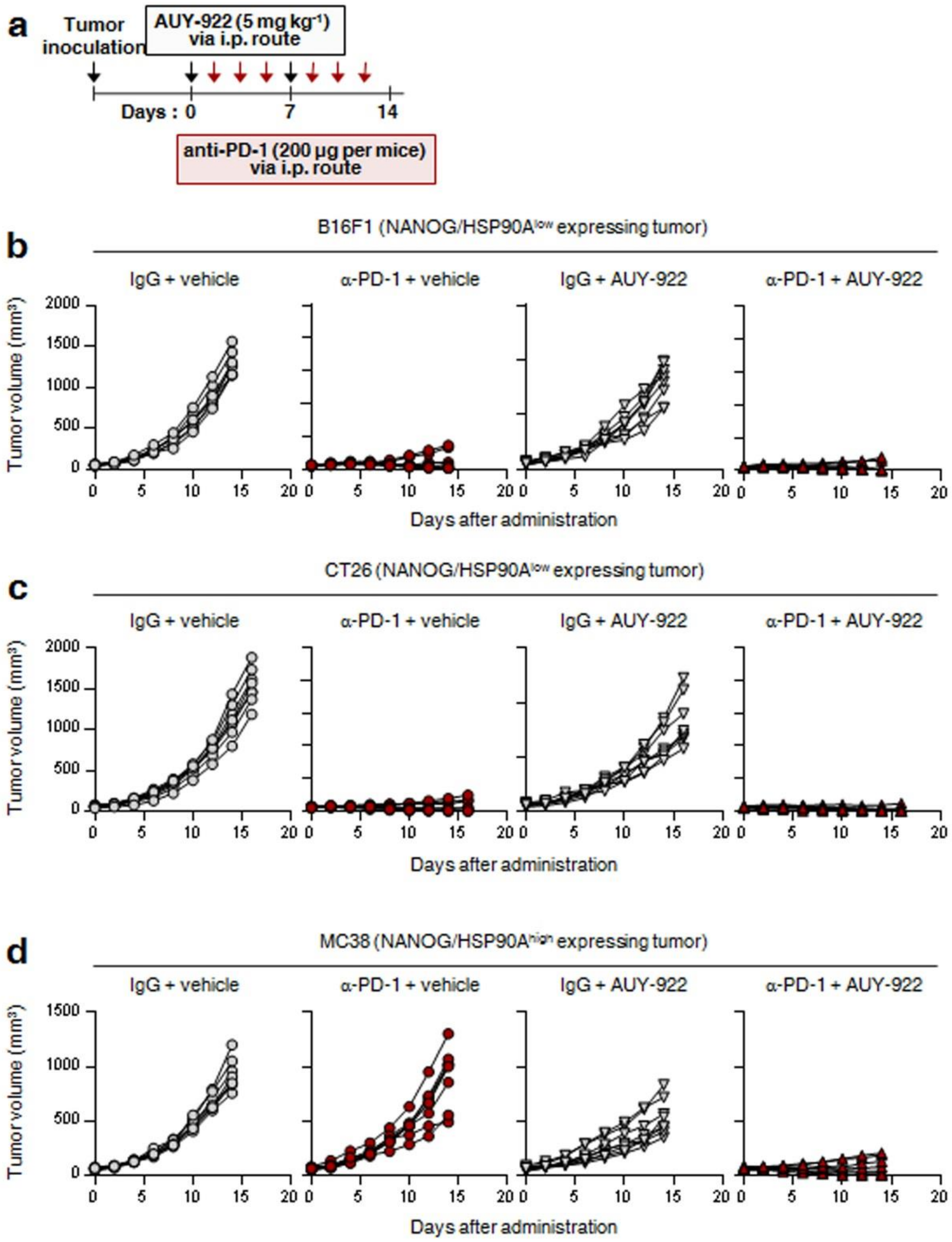

Supplementary Fig. 20 NANOG-HSP90A axis could be responsible for resistance to anti-PD-1 therapy. **a** Schematic of the therapy regimen in mice implanted with tumor cells. **b-d** Tumor growth in mice at indicated days after challenge. Tumor-bearing mice (N=10) administered vehicle or AUY-922, with or without treatment of anti-PD-1 antibody. For *in vivo* experiments, 10 mice from each group were used.

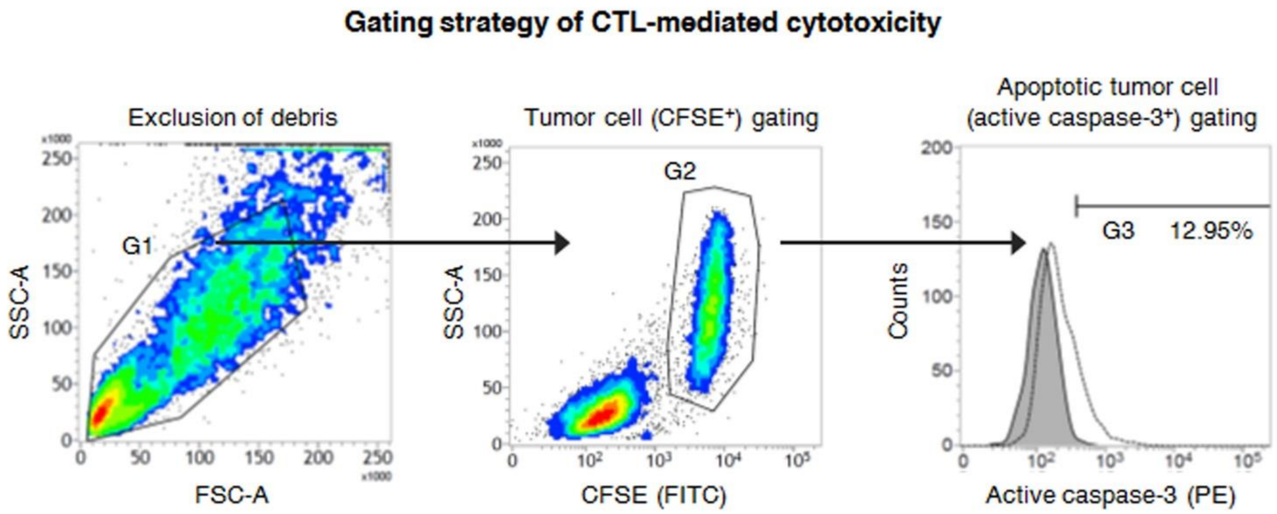

Supplementary Fig. 21 Gating strategy for identification of apoptotic tumor cells by CTL-mediated killing. Representative flow cytometry analysis showing the gating strategy. Population was initially gated on the basis of the forward side scatter characteristics and debris were eliminated, then tumor cells were selected, the CFSE$^+$ cells were gated in the plot CFSE (FITC) versus side scatter. Finally, the CFSE$^+$ active caspase-3$^+$ population was measured in the active caspase-3 (PE) histograms.

Supplementary Fig. 22 Uncropped blots

Fig. 1a

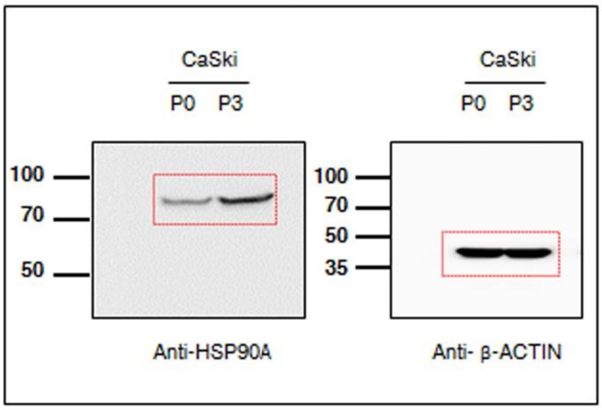

Fig. 2a

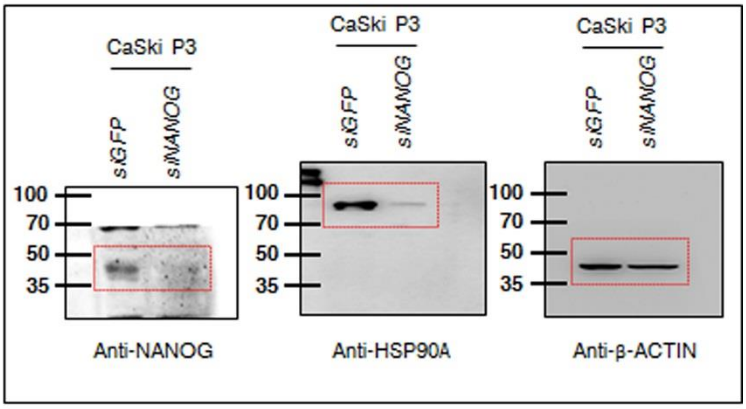

Fig. 2c

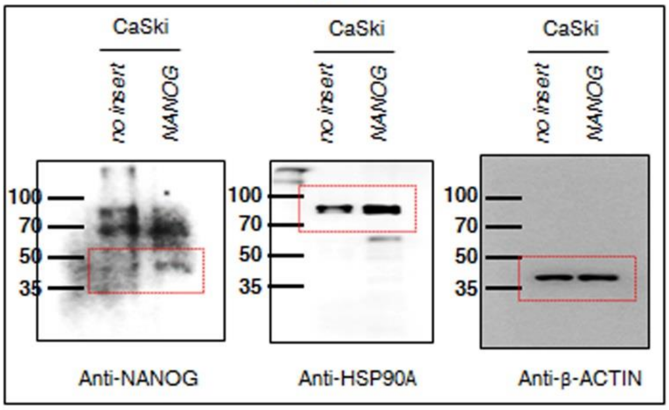

Fig. 2e

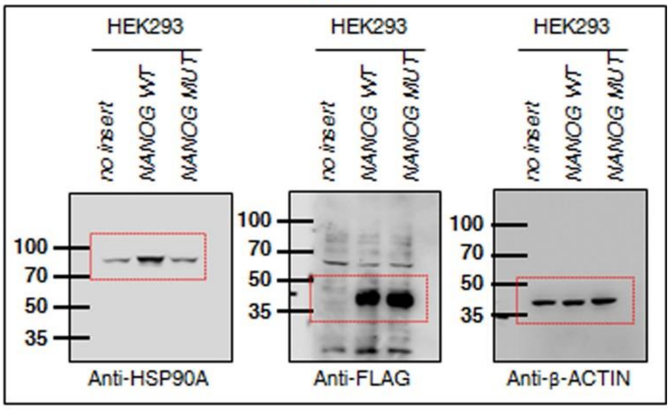

Fig. 3a

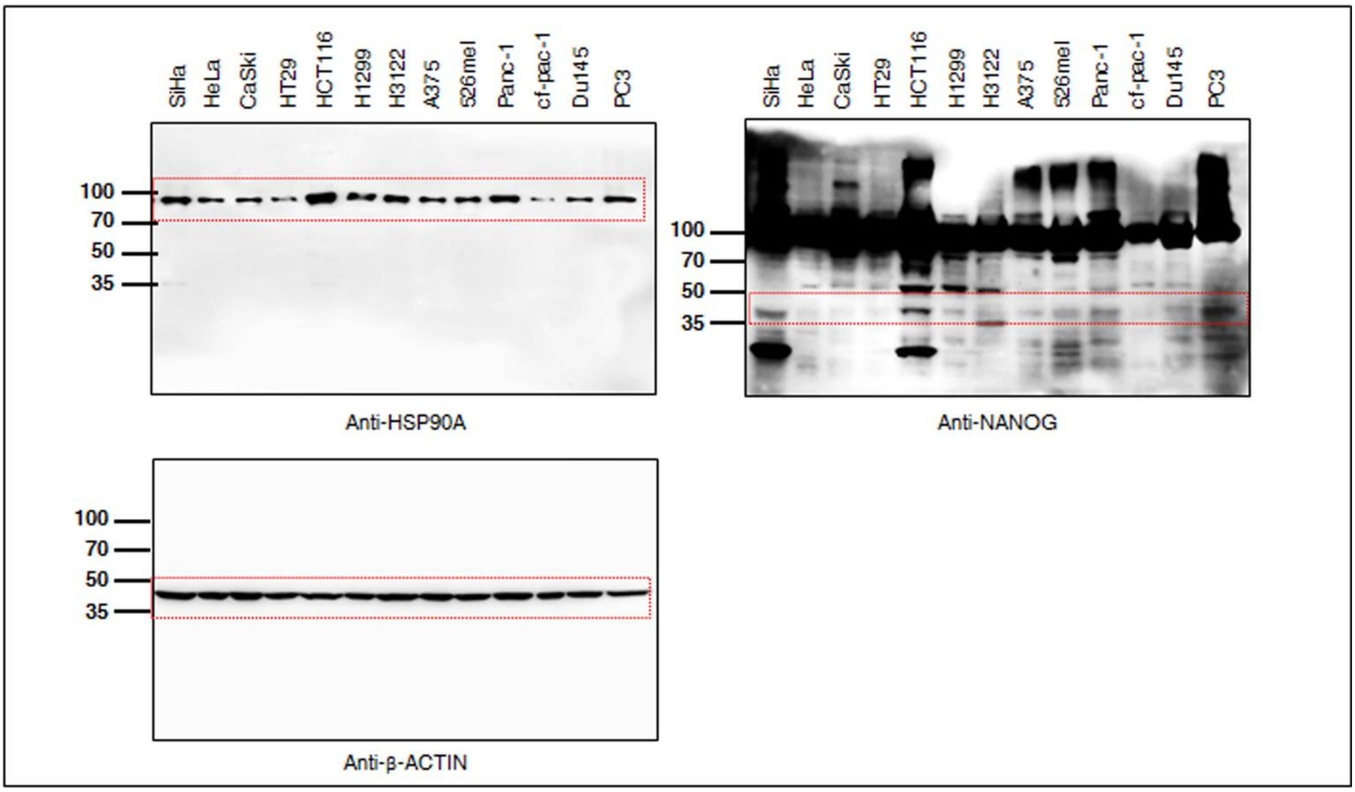

Supplementary Fig. 22 Uncropped blots (continued)

Fig. 4a

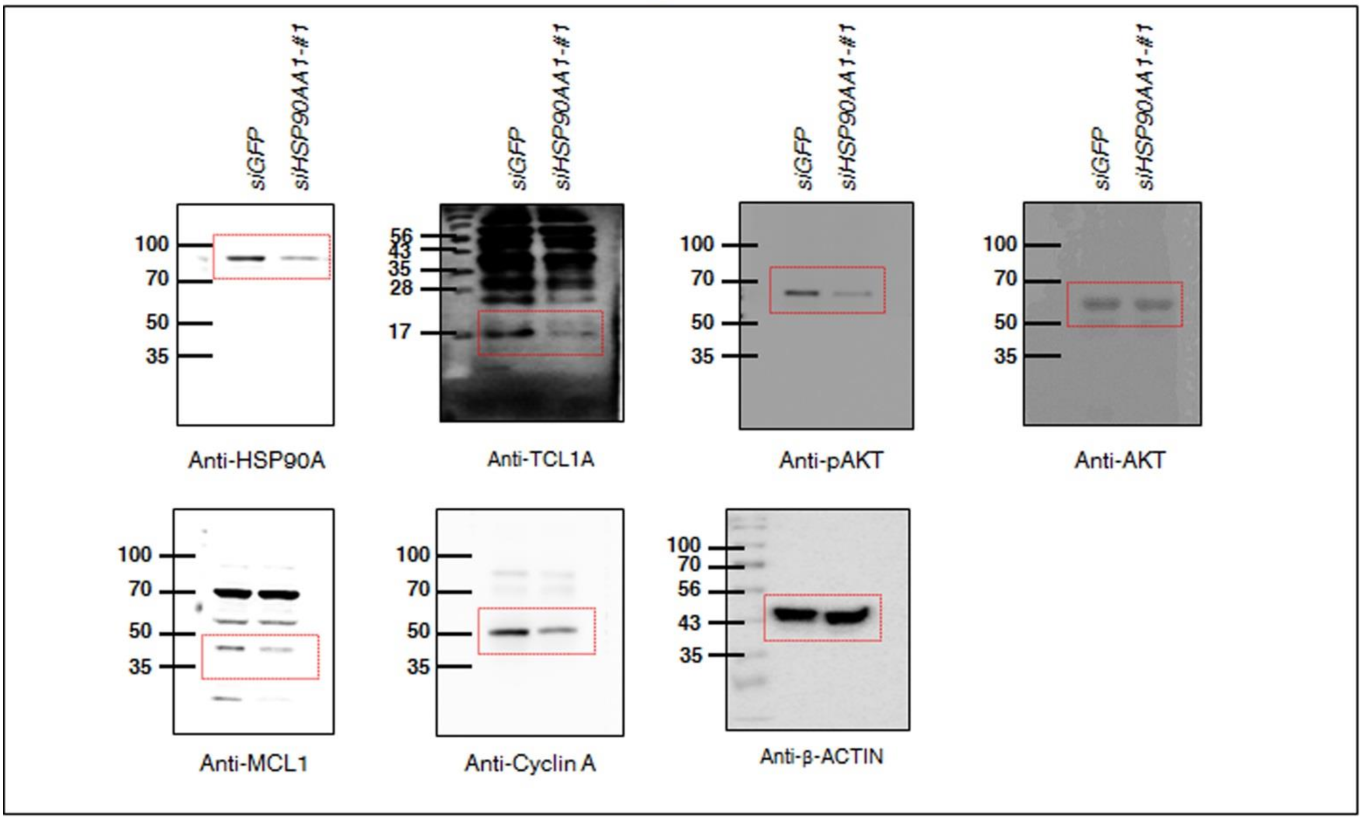

Fig. 4b

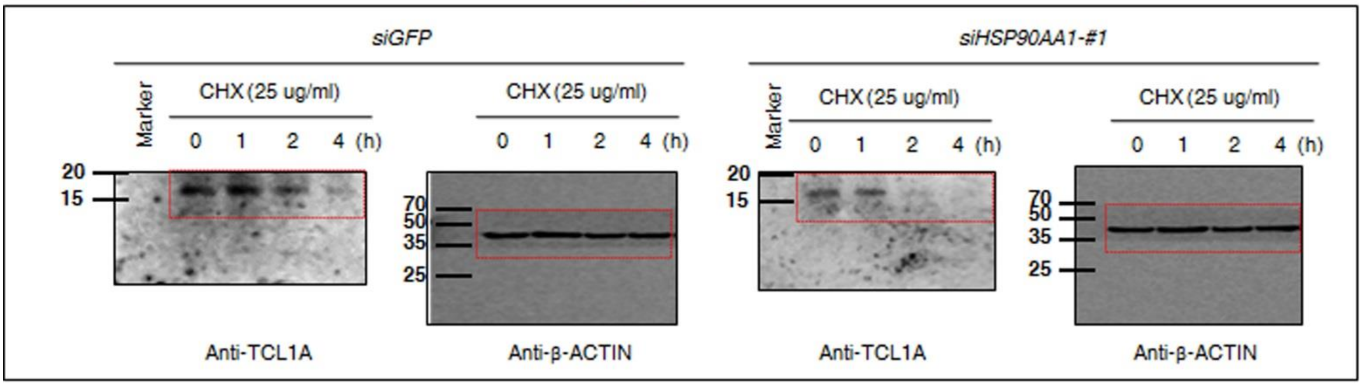

Fig. 4c

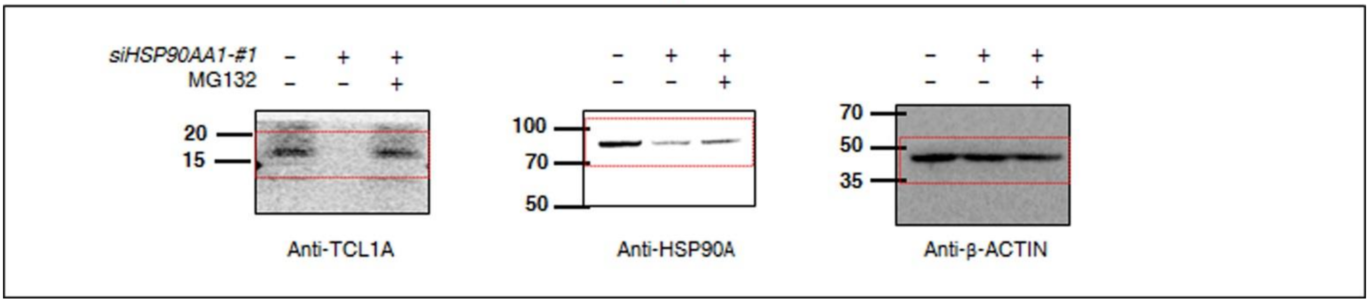

Supplementary Fig. 22 Uncropped blots (continued)

Fig. 4d

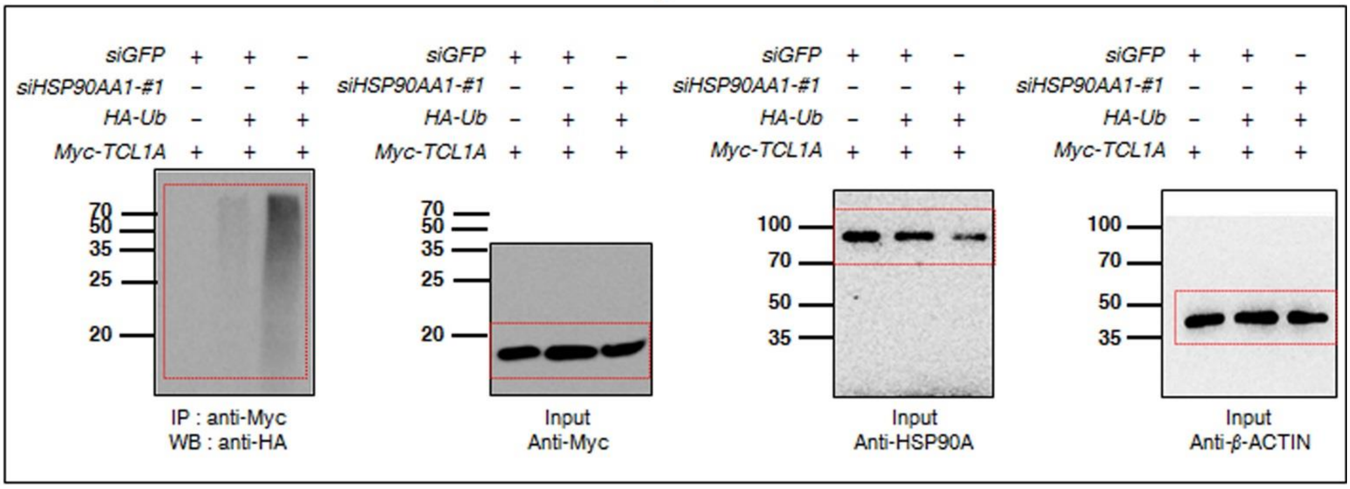

Fig. 4e

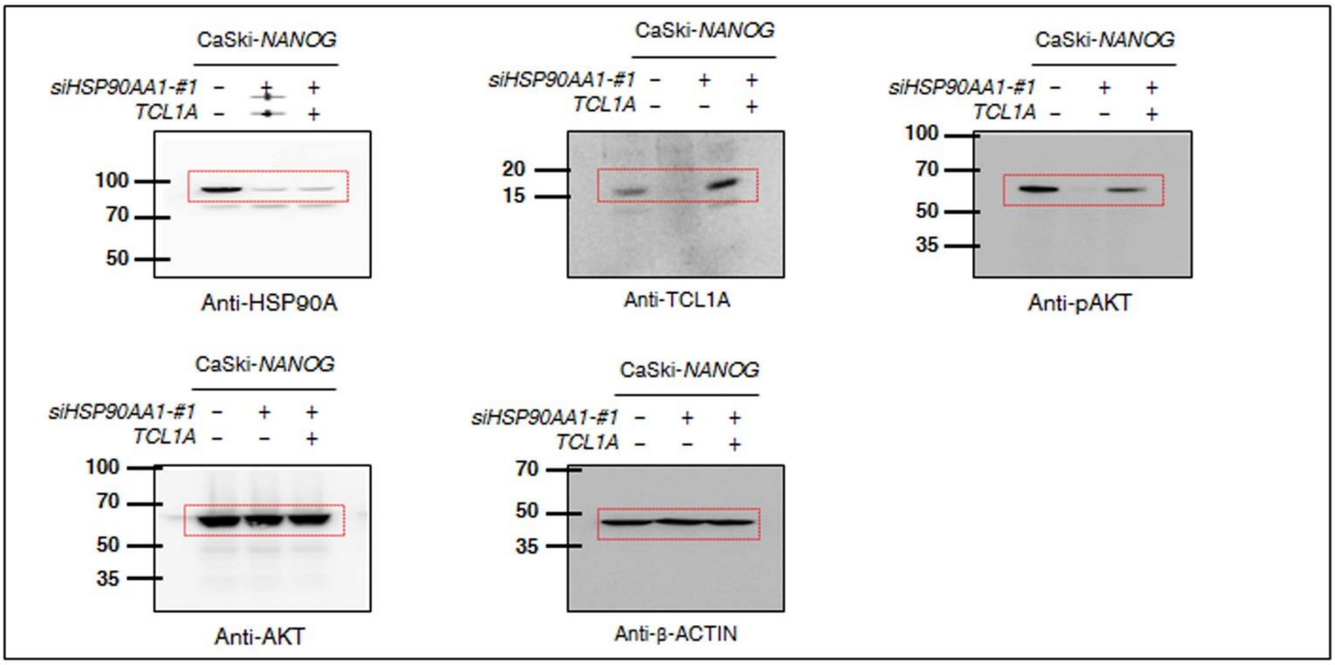

Fig. 4f

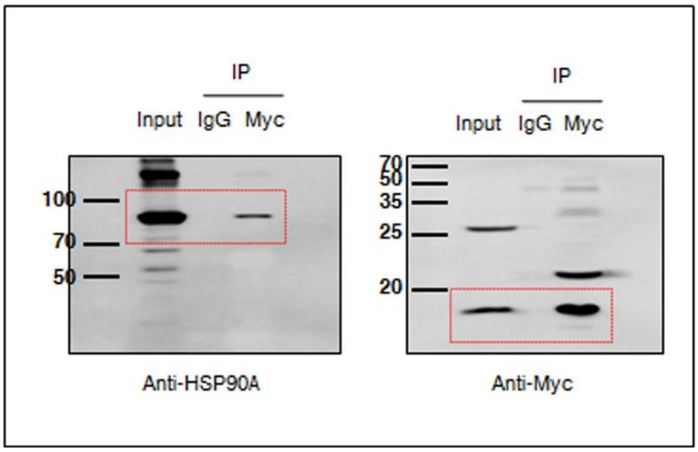

Supplementary Fig. 22 Uncropped blots (continued)

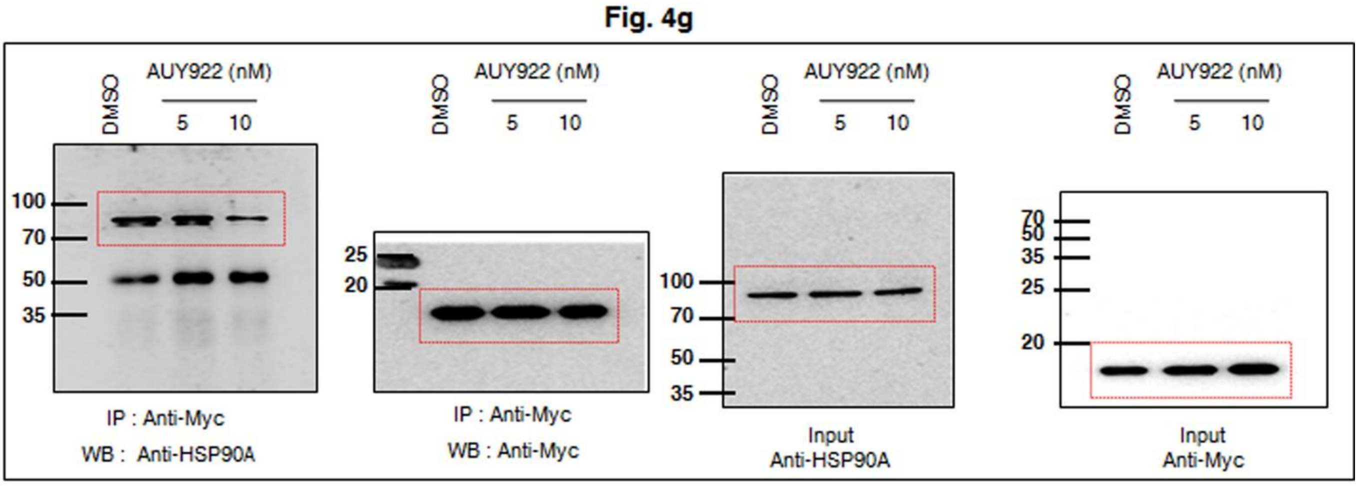

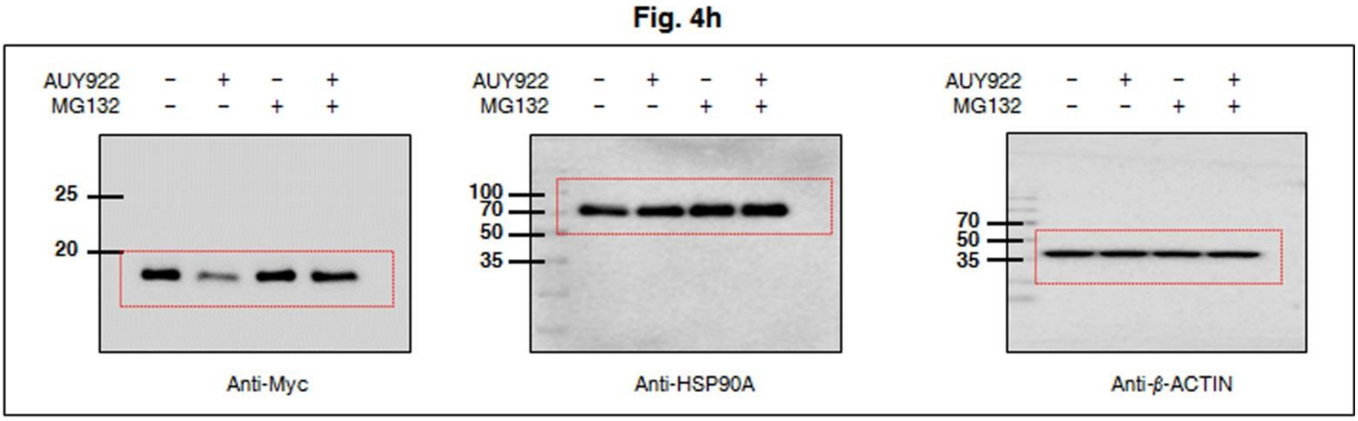

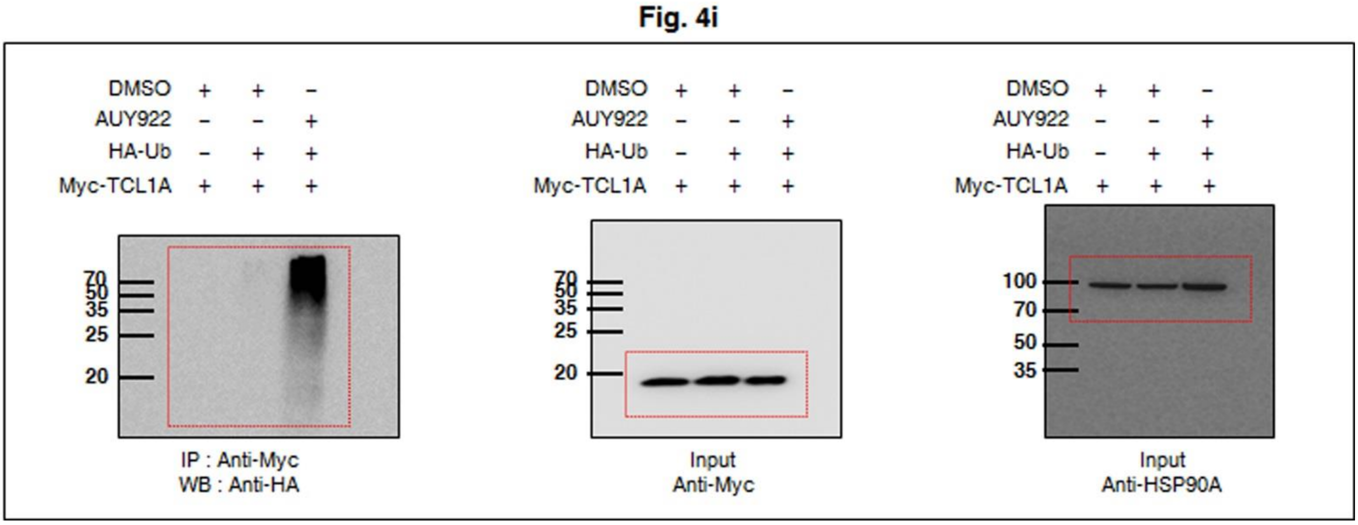

Supplementary Fig. 22 Uncropped blots (continued)

Fig. 5a

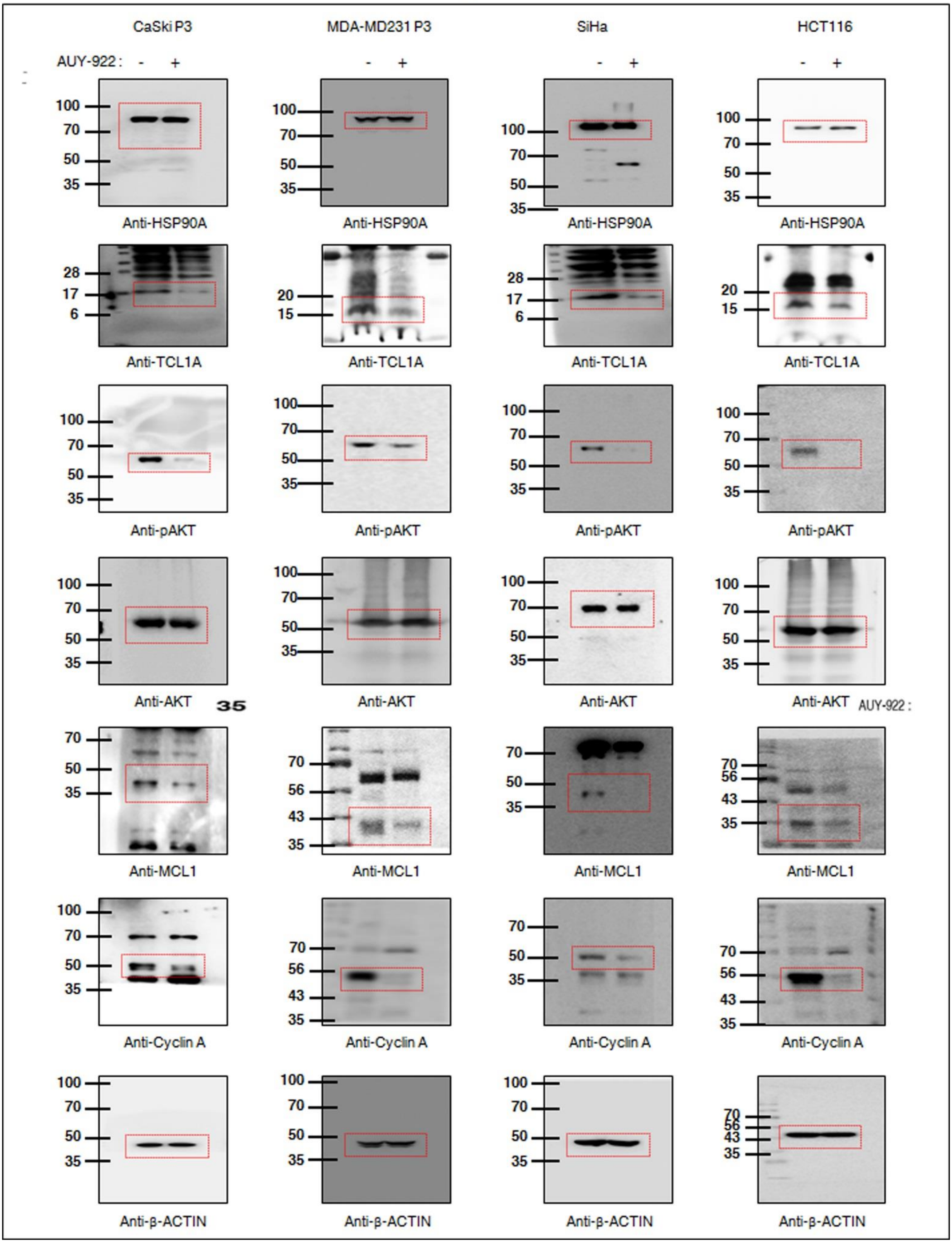

Supplementary Fig. 22 Uncropped blots (continued)

Fig. 6e

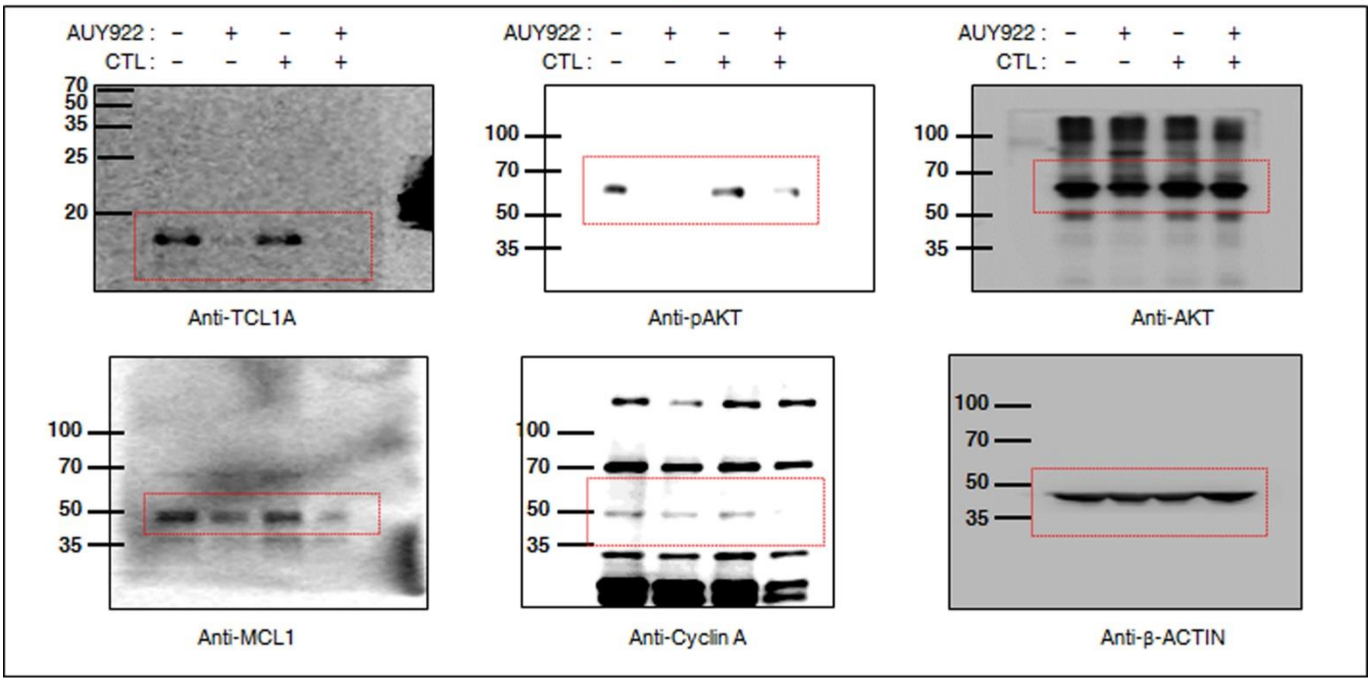

Supplementary Fig. 22 Uncropped blots (continued)

Supplementary Fig. 1

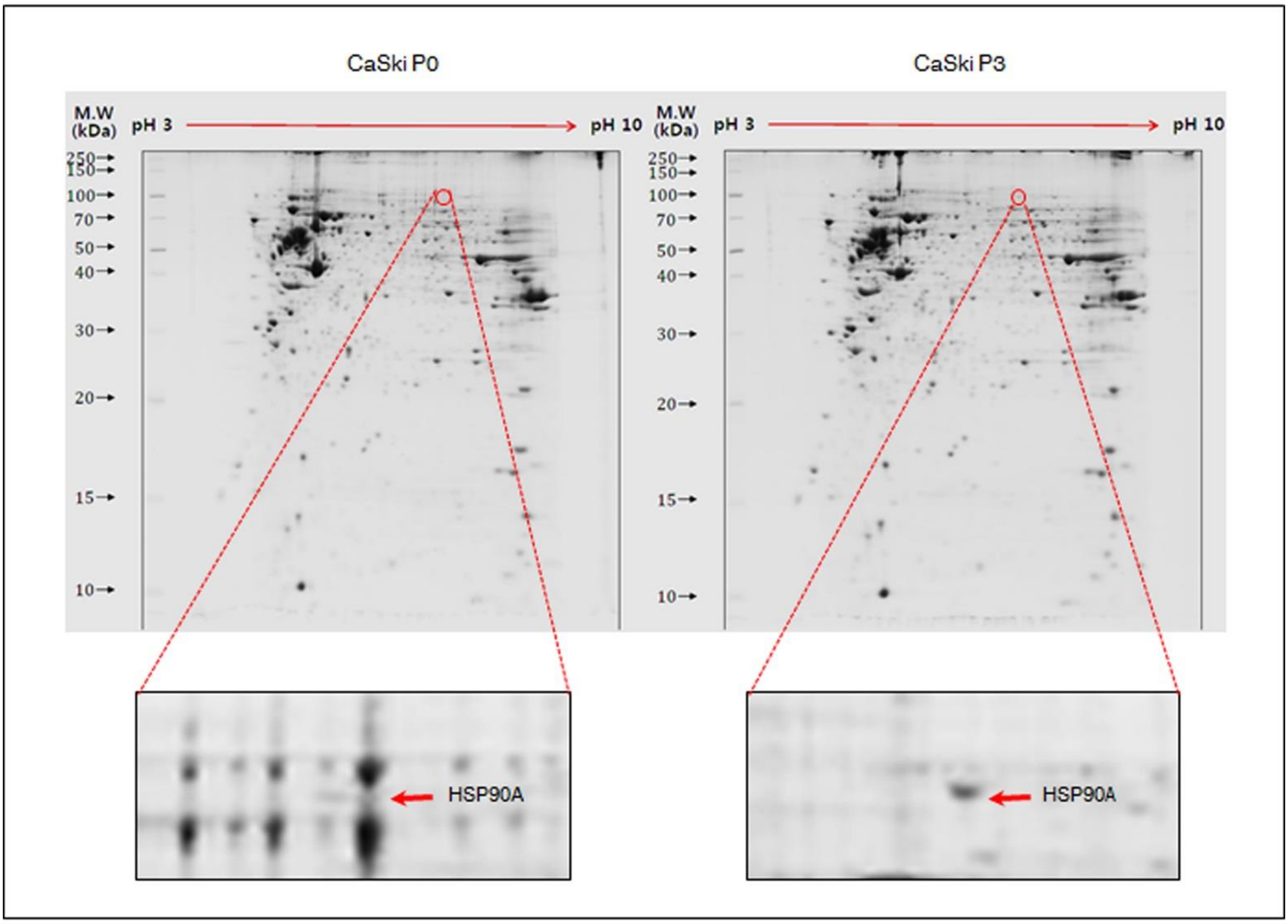

Supplementary Fig. 3a

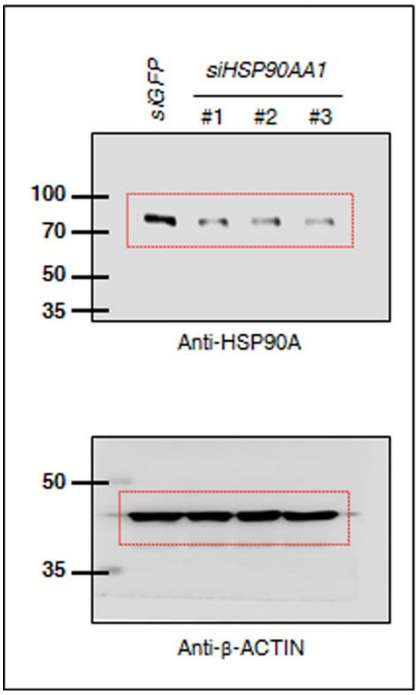

Supplementary Fig. 4a

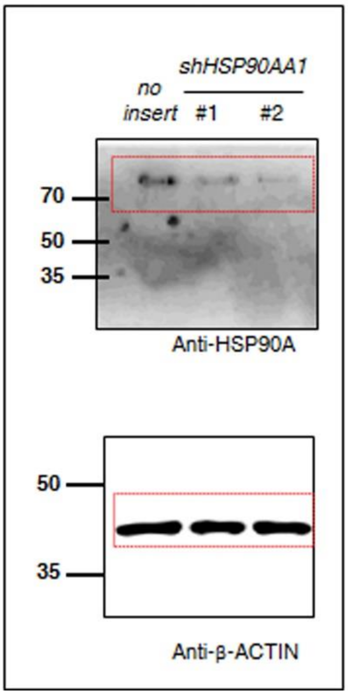

Supplementary Fig. 5b

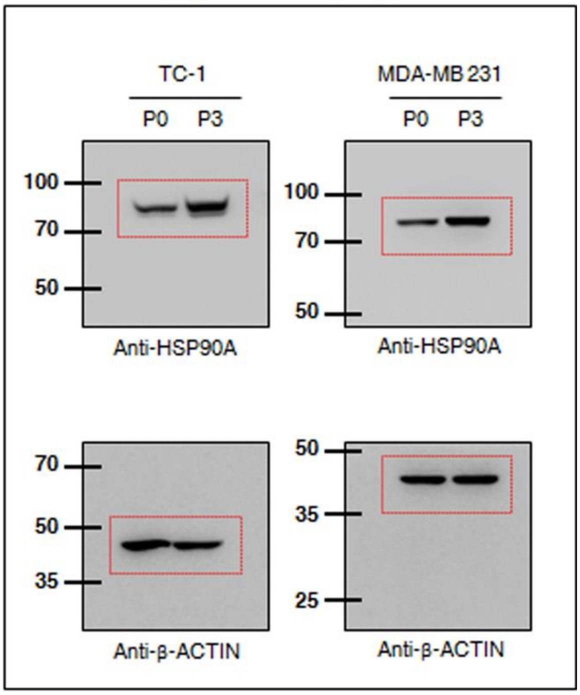

Supplementary Fig. 22 Uncropped blots (continued)

Supplementary Fig. 12a

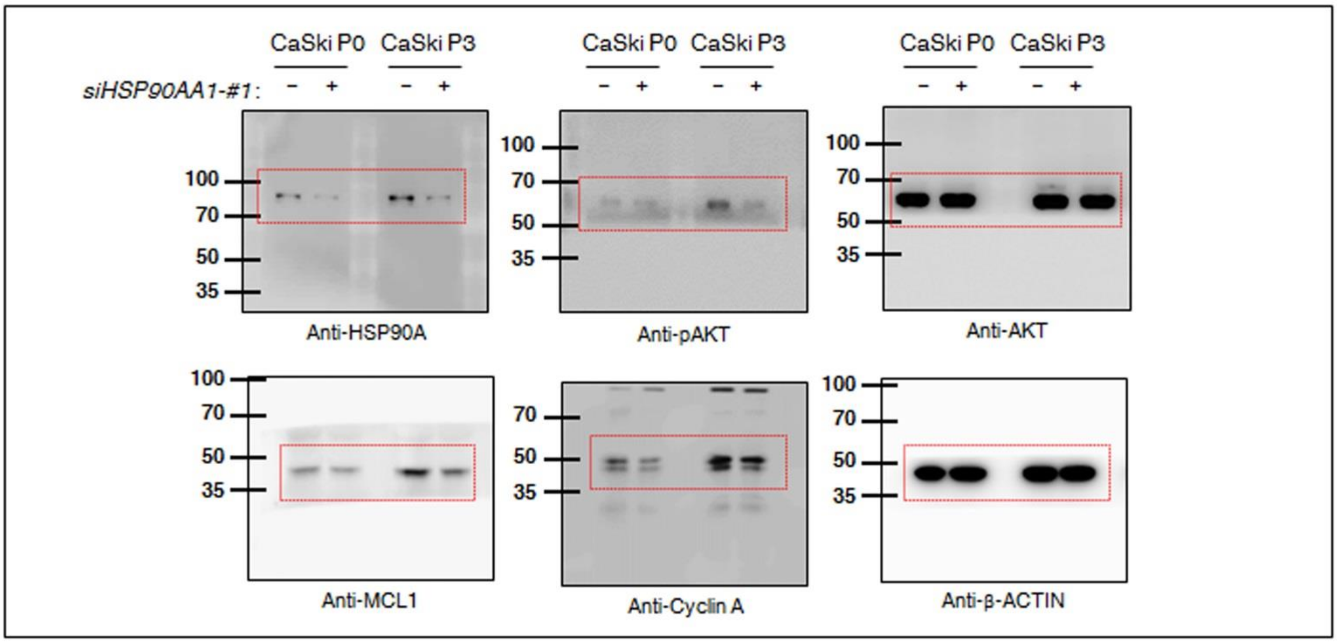

Supplementary Fig. 12b

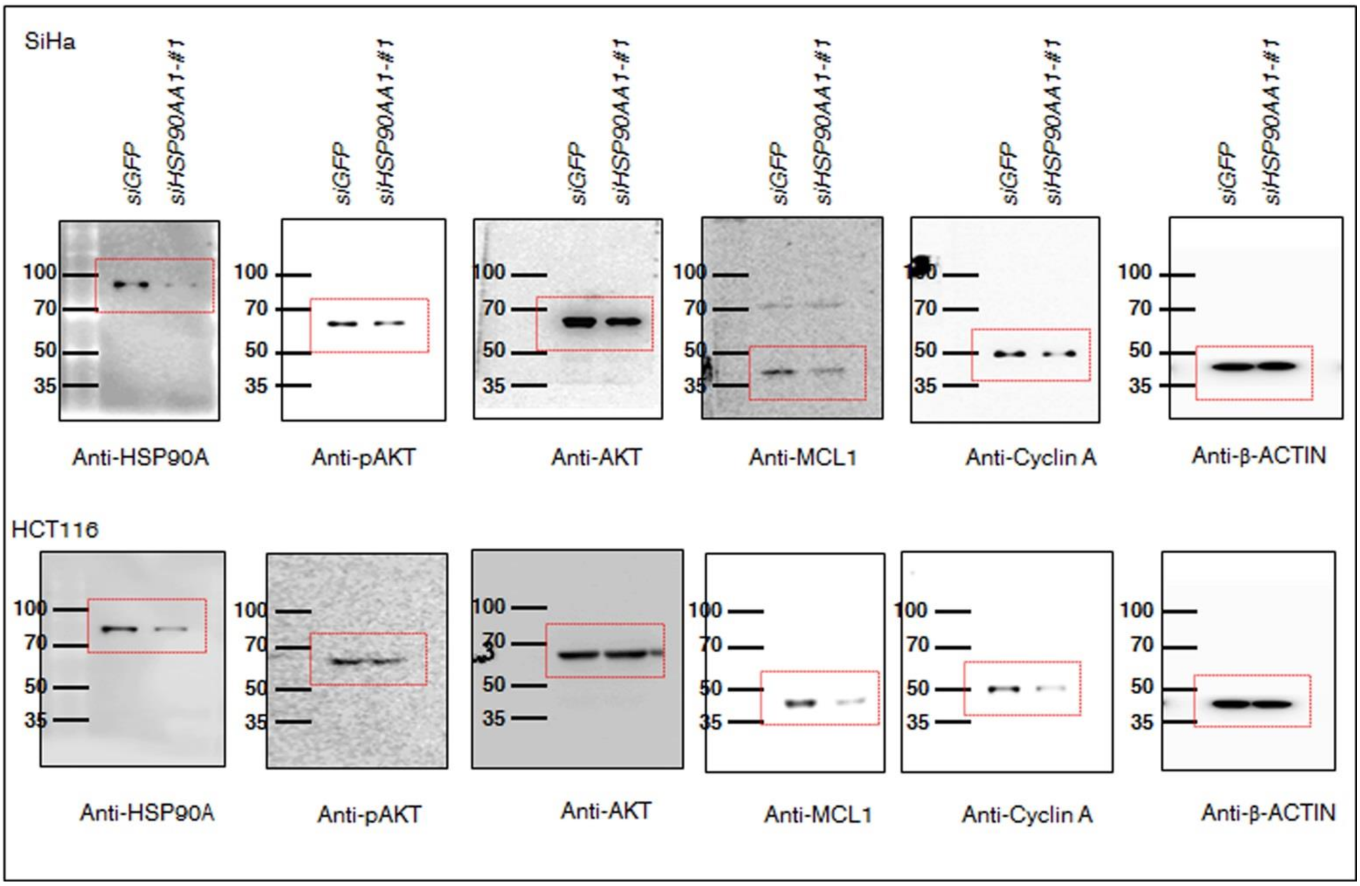

Supplementary Fig. 22 Uncropped blots (continued)

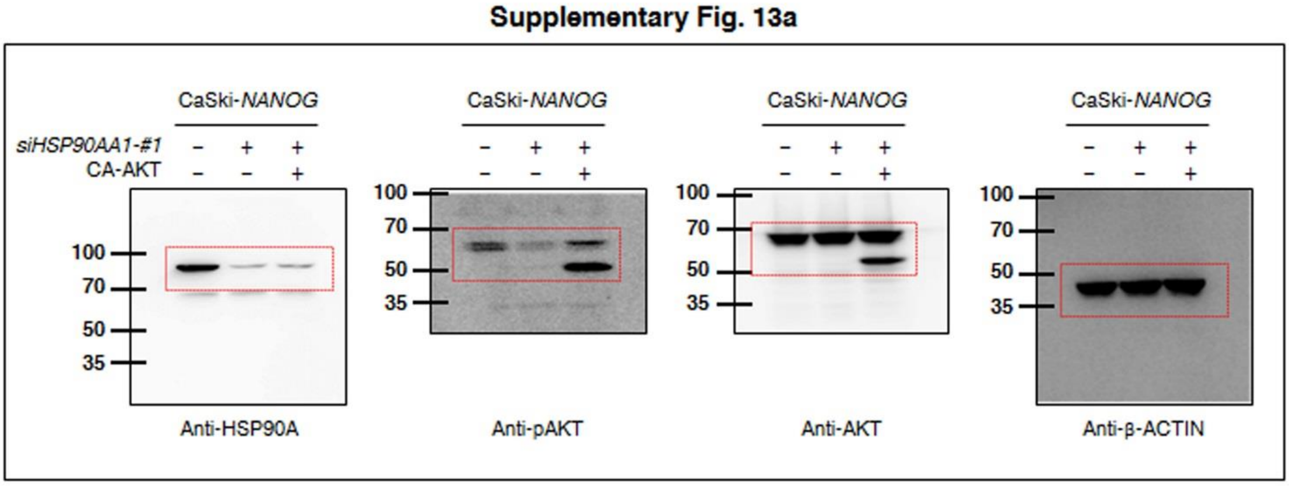

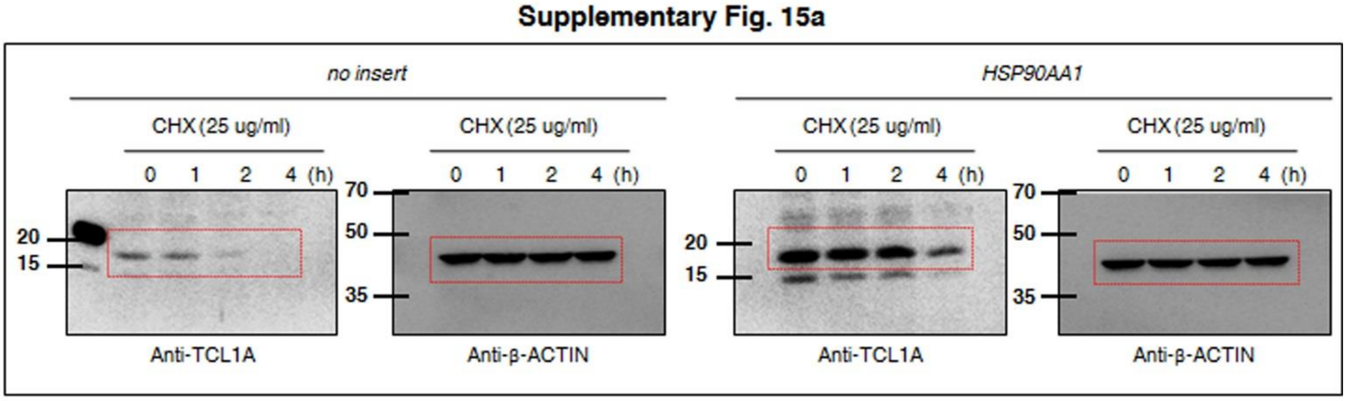

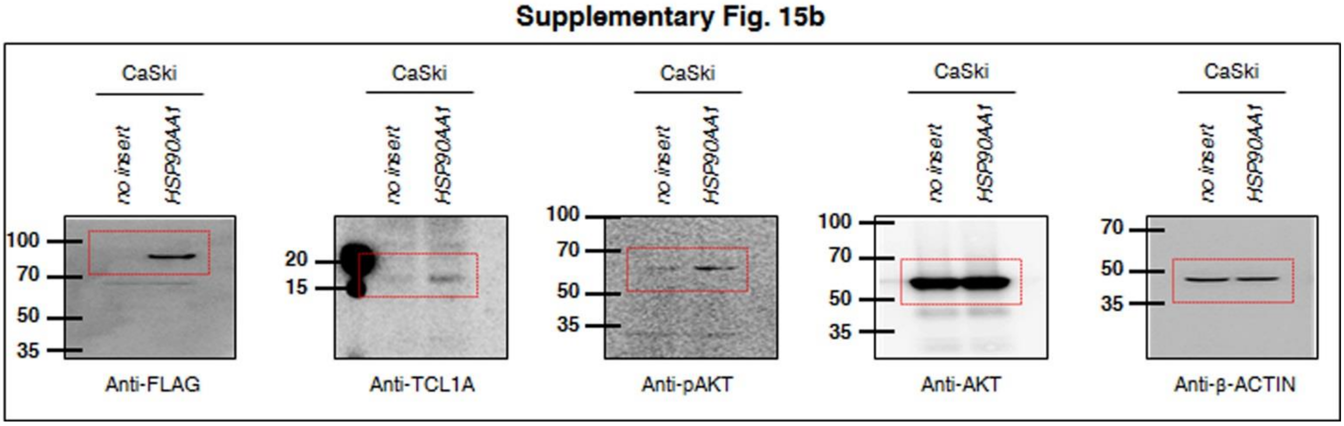

Supplementary Fig. 22 Uncropped blots (continued)

Supplementary Fig. 18a

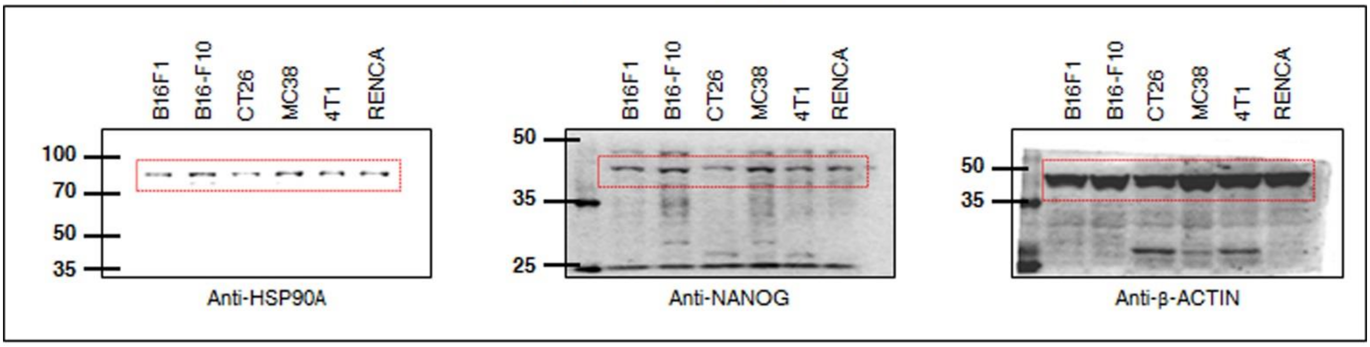

Supplementary Fig. 18b

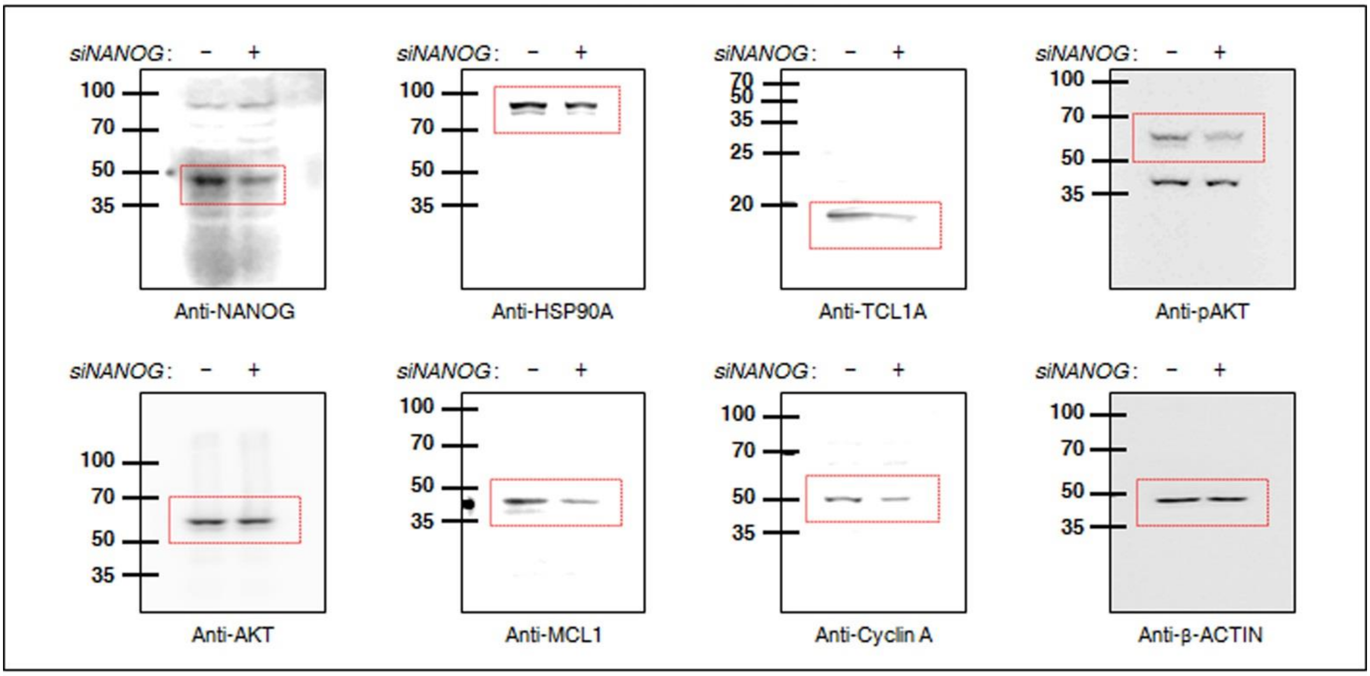

Supplementary Fig. 18c

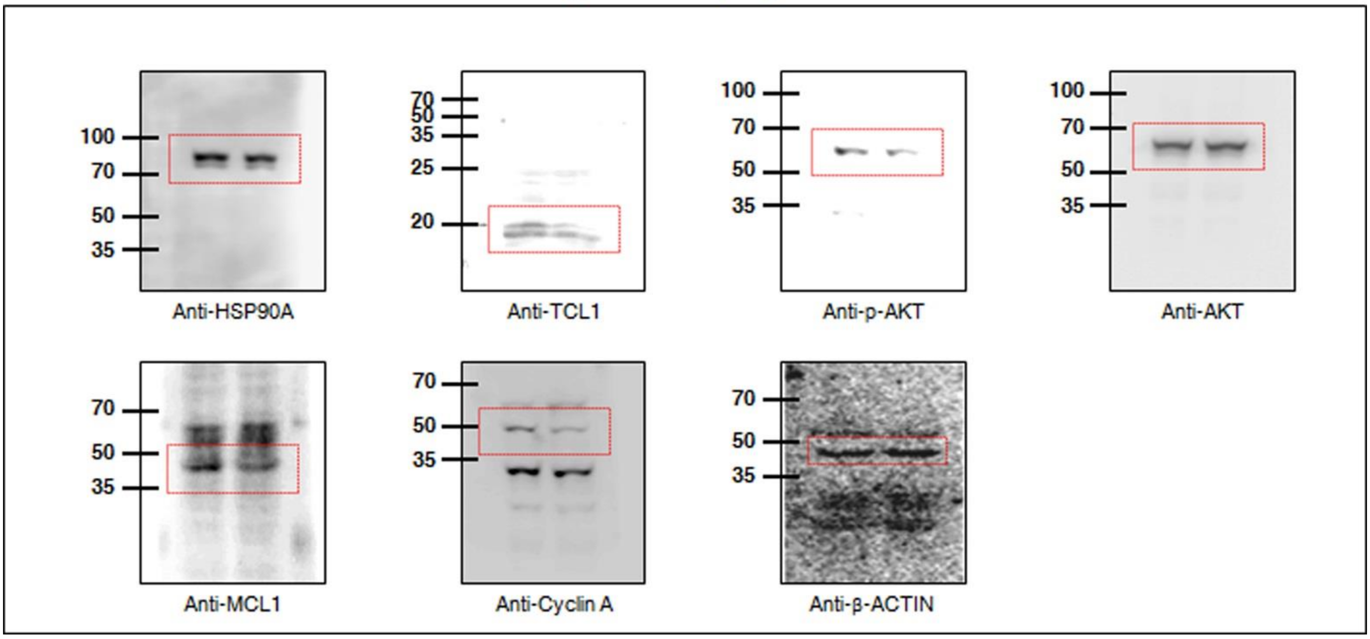

Supplementary Table 1 Clinicopathologic significance of HSP90A level in human cervical neoplasias.

	HSP90A		
	No.	Mean IHC score (95% CI)	*p*-value
			<0.001
Normal	328	13 (11-16)	
Low grade CIN	65	111 (101-122)	
High grade CIN	160	156 (147-165)	
Cancer	151	189 (179-200)	
**Age**			*0.279*
≤ 50	87	185 (171-198)	
> 50	64	196 (181-211)	
**FIGO stage**			*0.410*
I - IIA	112	187 (175-200)	
IIB - IV	39	196 (179-212)	
**Cell type**			*0.511*
SCC	126	188 (177-200)	
Others	25	196 (176-215)	
**Tumor grade**			*0.189*
Well+Moderate	99	196 (184-208)	
Poor	45	181 (161-201)	
**Tumor size**			***0.028***
< 4 cm	101	182 (169-194)	
≥ 4 cm	50	205 (189-222)	
**LN metastasis**			*0.829*
Negative	92	190 (176-204)	
Positive	29	187 (169-206)	
**Chemoradiation response**			*0.068*
Good	44	212 (199-225)	
Bad	20	188 (162-214)	

SCC, squamous cell carcinoma; FIGO, International Federation of Gynecology and Obstetrics; LN metastasis, Lymph node metastasis. Protein expression was determined through analysis of an immunohistochemically stained tissue array, as described in the materials and methods section. The statistical significance of the differences in the protein expressions in the different groups was calculated by the Mann–Whitney U test. Chi-square test was used to assess associations between protein expressions and tumor size. Results with two-tailed *p*-values less than 0.05 were considered statistically significant.

Supplementary Table 2 Univariate and multivariate analyses of disease-free survival in cervical cancer patients.

Variables	Univariate analysis		Multivariate analysis	
	Hazard ratio [95%CI]	*p*-value	Hazard ratio [95%CI]	*p*-value
Age (>50)	1.39 [0.70 - 2.75]	0.347	NA	
FIGO stage (> IIB)	5.21 [2.61 - 10.39]	<0.001	2.51 [0.95 - 6.66]	0.063
Cell type (non-SCC)	1.70 [0.76 - 3.79]	0.188	NA	
Grade (poor)	1.52 [0.75 - 3.06]	0.237	NA	
Tumor size (>4 cm)	1.75 [0.87 - 3.49]	0.111	NA	
LN metastasis	4.04 [1.83 - 8.92]	0.001	1.85 [0.69 - 4.94]	0.218
SCC Ag$^+$	1.51 [0.71 - 3.20]	0.276	NA	
NANOG$^+$ [a]	4.59 [1.99 - 10.62]	<0.001	3.89 [1.41 - 10.69]	0.008
HSP90A$^+$ [b]	3.53 [0.83 - 14.93]	0.086	NA	
NANOG$^+$/HSP90A$^+$	3.64 [1.78 - 7.43]	<0.001	3.27 [1.30 - 8.24]	0.012

[a]cut-off value of NANOG$^+$ is over 160 of IHC score; [b]cut-off of HSP90A$^+$ is over 127 of IHC score; CI, confidence interval; FIGO, International Federation of Gynecology and Obstetrics; LN, lymph node; NA, not applicable. Survival curves were calculated using the Kaplan-Meier method and the difference between the survival curves was calculated by the log-rank test.

Supplementary Table 3 Univariate and multivariate analyses of overall survival in cervical cancer patients.

Variables	Univariate analysis		Multivariate analysis	
	Hazard ratio [95%CI]	*p*-value	Hazard ratio [95%CI]	*p*-value
Age (>50)	1.15 [0.47 - 2.81]	0.759	NA	
FIGO stage (> IIB)	3.90 [1.59 - 9.56]	0.003	3.24 [1.28 - 8.21]	0.013
Cell type (non-SCC)	3.23 [1.27 - 8.18]	0.013	3.24 [1.27 - 8.30]	0.014
Grade (poor)	1.99 [0.81 - 4.91]	0.133	NA	
Tumor size (> 4 cm)	1.32 [0.52 - 3.35]	0.554	NA	
LN metastasis	2.09 [0.70 - 6.21]	0.182	NA	
SCC Ag^+	2.47 [0.94 - 6.51]	0.066	NA	
$NANOG^{+}$ [a]	4.70 [1.57 - 14.11]	0.006	4.00 [1.31 - 12.25]	0.015
$HSP90A^{+}$ [b]	4.45 [0.57 - 34.39]	0.152	NA	
$NANOG^+$/$HSP90A^+$	4.08 [1.61 - 10.36]	0.003	4.03 [1.53 - 10.55]	0.005

[a]cut-off value of $NANOG^+$ is over 160 of IHC score; [b]cut-off of $HSP90A^+$ is over 127 of IHC score; CI, confidence interval; FIGO, International Federation of Gynecology and Obstetrics; LN, lymph node; NA, not applicable Survival curves were calculated using the Kaplan-Meier method and the difference between the survival curves was calculated by the log-rank test.

Supplemental Table 4 Primer sequences used for quantitative RT-PCR.

Assay	Target	Forward (5 '- 3')	Reverse (5' - 3')	Amplicon (bp)
quantitative RT-PCR	HSP90AA1	TGGACAGCAAACATGGAGAG	AGACAGGAGCGCAGTTTCAT	204
	HSP90AB1	AGAAATTGCCCAACTCATGTCC	ATCAACTCCCGAAGGAAAATCTC	75
	HSP90B1	CCAGTTTGGTGTCGGTTTCTAT	CTGGGTATCGTTGTTGTGTTTTG	81
	TCL1A	GCCTGGGAGAAGTTCGTGTA	TCAGTCATCTGGCAGCAGC	288
	β-ACTIN	CATGTACGTTGCTATCCAGGC	CTCCTTAATGTCACGCACGAT	250
	Hsp90aa1	AATTGCCCAGTTAATGTCCTTGA	CGTCCGATGAATTGGAGATGAG	89
	Hsp90ab1	GTCCGCCGTGTGTTCATCAT	GCACTTCTTGACGATGTTCTTGC	168
	Hsp90b1	TCGTCAGAGCTGATGATGAAGT	GCGTTTAACCCATCCAACTGAAT	136
	β-Actin	GGCTGTATTCCCCTCCATCG	CCAGTTGGTAACAATGCCATGT	154
