# Supplementary material for: HSP90A inhibition promotes anti-tumor immunity by reversing multi-modal resistance and stem-like property of immune-refractory tumors
Source: Nat Commun. 2020 Jan 28;11:562. doi: 10.1038/s41467-019-14259-y (PMC6987099; doi:10.1038/s41467-019-14259-y)
Supplement: Supplementary file 2 — Reporting Summary [file 41467_2019_14259_MOESM2_ESM.pdf]

nature research

Corresponding author(s): Tae Woo Kim

Last updated by author(s): Dec 12, 2019

Reporting Summary

Nature Research wishes to improve the reproducibility of the work that we publish. This form provides structure for consistency and transparency in reporting. For further information on Nature Research policies, see Authors & Referees and the Editorial Policy Checklist .

Statistics

For all statistical analyses, confirm that the following items are present in the figure legend, table legend, main text, or Methods section.

n/a	Confirmed	
☐	☒	The exact sample size (*n*) for each experimental group/condition, given as a discrete number and unit of measurement
☐	☒	A statement on whether measurements were taken from distinct samples or whether the same sample was measured repeatedly
☐	☒	The statistical test(s) used AND whether they are one- or two-sided<br>*Only common tests should be described solely by name; describe more complex techniques in the Methods section.*
☐	☒	A description of all covariates tested
☐	☒	A description of any assumptions or corrections, such as tests of normality and adjustment for multiple comparisons
☐	☒	A full description of the statistical parameters including central tendency (e.g. means) or other basic estimates (e.g. regression coefficient) AND variation (e.g. standard deviation) or associated estimates of uncertainty (e.g. confidence intervals)
☐	☒	For null hypothesis testing, the test statistic (e.g. *F*, *t*, *r*) with confidence intervals, effect sizes, degrees of freedom and *P* value noted<br>*Give P values as exact values whenever suitable.*
☒	☐	For Bayesian analysis, information on the choice of priors and Markov chain Monte Carlo settings
☒	☐	For hierarchical and complex designs, identification of the appropriate level for tests and full reporting of outcomes
☐	☒	Estimates of effect sizes (e.g. Cohen's *d*, Pearson's *r*), indicating how they were calculated

Our web collection on statistics for biologists contains articles on many of the points above.

Software and code

Policy information about availability of computer code

Data collection	Cancer Genome Atlas (TCGA) portal (https://tcga-data.nci.nih.gov/tcga/)<br>GSE91061
Data analysis	GraphPad Prism Ver. 6.0 (GraphPad Software, Inc., San Deigo, CA, USA) , TF-LOF Expression from GEO (Gene Expression Omnibus)

For manuscripts utilizing custom algorithms or software that are central to the research but not yet described in published literature, software must be made available to editors/reviewers. We strongly encourage code deposition in a community repository (e.g. GitHub). See the Nature Research guidelines for submitting code & software for further information.

Data

Policy information about availability of data

All manuscripts must include a data availability statement. This statement should provide the following information, where applicable:

- Accession codes, unique identifiers, or web links for publicly available datasets
- A list of figures that have associated raw data
- A description of any restrictions on data availability

RNA-seq datasets from samples of 32 cancer types are available in the Cancer Genome Atlas (TCGA) portal (https://tcga-data.nci.nih.gov/tcga/).
To investigate the clinical relevance in patients treated with anti-PD-1 therapy, we used the published datasets (GSE91061, https://www.ncbi.nlm.nih.gov/geo/query/acc.cgi?acc=GSE91061)

Field-specific reporting

Please select the one below that is the best fit for your research. If you are not sure, read the appropriate sections before making your selection.

Life sciences study design

All studies must disclose on these points even when the disclosure is negative.

Sample size	No sample-size calculation was performed. Mice numbers were determined by experience in Xenografts study.
Data exclusions	No data were excluded.
Replication	All data are representative of at least 3 separate experiments. All attempt at replication were successful
Randomization	For in vivo treatment experiments, tumor-bearing mice were subjected to caliper measurements. Animals with comparable tumor sizes were randomized into treatment groups; this prevented outcomes from being influenced by initial differences in tumor burden.
Blinding	To give different treatments to different experimental groups, the investigators were not blinded in Xenografts study.

Reporting for specific materials, systems and methods

We require information from authors about some types of materials, experimental systems and methods used in many studies. Here, indicate whether each material, system or method listed is relevant to your study. If you are not sure if a list item applies to your research, read the appropriate section before selecting a response.

Materials & experimental systems

n/a	Involved in the study	
☐	☒	Antibodies
☐	☒	Eukaryotic cell lines
☒	☐	Palaeontology
☐	☒	Animals and other organisms
☐	☒	Human research participants
☒	☐	Clinical data

Methods

n/a	Involved in the study	
☒	☐	ChIP-seq
☐	☒	Flow cytometry
☒	☐	MRI-based neuroimaging

Antibodies

Antibodies used	Details about all antibodies used in this study are provided in the Materials and Methods section of the manuscript.
Validation	Validation statements for antibodies can be found on their corresponding manufacturer websites. Validation in our own samples has been confirmed by Western blot detection of bands at the predicted size.

Eukaryotic cell lines

Policy information about cell lines

Cell line source(s)	CaSki, MDA-MB231, SiHa, HCT116 and HEK293 cell lines were purchased from American Type Culture Collection (ATCC, Manassas, VA, USA). All cell lines were obtained between 2010 and 2014. Generation of the immune edited CaSki P3 cell line is described in Clinical cancer research 2015;21(6):1438-46. Generation of the immune edited MDA-MB P3 and CaSki-NANOG cell line is described in Cancer research 2017;77(18):5039-53.
Authentication	The identities of cell lines were confirmed by short tandem repeat (STR) profiling by IDEXX Laboratories Inc. and used within 6 months for testing.
Mycoplasma contamination	All cell lines were tested for mycoplasma using Mycoplasma Detection Kit (Thermo Fisher Scientific, San Jose, CA, USA).
Commonly misidentified lines (See ICLAC register)	N/A

Animals and other organisms

Policy information about studies involving animals; ARRIVE guidelines recommended for reporting animal research

Laboratory animals	6- to 8- week-old female NOD/SCID or C57BL/6 mice were used in this project.
Wild animals	This study did not involve wild animals.

Field-collected samples	The study did not involve samples collected from the field.
Ethics oversight	All mice were maintained and handled under the protocol approved by the Korea University Institutional Animal Care and Use Committee (KOREA-2017-0141). All animal procedures were performed in accordance with recommendations for the proper use and care of laboratory animals.

Note that full information on the approval of the study protocol must also be provided in the manuscript.

Human research participants

Policy information about studies involving human research participants

Population characteristics	The study subjects were compromised of 169 cervical cancers and 314 cervical intraepithelial neoplasia (CIN) patients who underwent surgical resection in Gangnam Severance Hospital between 1996 and 2010.
Recruitment	Tissue samples were collected from patients who had signed informed consent form.
Ethics oversight	This study was approved by the Institutional Review Board of Gangnam Severance Hospital (Seoul, South Korea), and all procedures were conducted in accordance with the guidelines of the Declaration of Helsinki.

Note that full information on the approval of the study protocol must also be provided in the manuscript.

Flow Cytometry

Plots

Confirm that:

- [x] The axis labels state the marker and fluorochrome used (e.g. CD4-FITC).
- [x] The axis scales are clearly visible. Include numbers along axes only for bottom left plot of group (a 'group' is an analysis of identical markers).
- [x] All plots are contour plots with outliers or pseudocolor plots.
- [x] A numerical value for number of cells or percentage (with statistics) is provided.

Methodology

Sample preparation	For CTL-mediated apoptosis assay, CaSki and MDA-MB231 cells were labeled with CFSE (10 µM, Molecular Probes, Eugene, OR) in DMEM supplemented with 0.1% FBS. The CFSE-labeled CaSki cells were pulsed with MART1 peptide (10 µg/ml) for 1 hour. The CFSE-labeled MDA-MB231 cells and CaSki cells were mixed with cognate MART-1 or control noncognate NY-ESO1–specific CD8+ CTLs at a 1:1 ratio and incubated for 4 hours at 37°C. For Granzyme B-mediated apoptosis assay, recombinant human granzyme B (Enzo Life Sciences) was mixed with BioPorter Reagent (Sigma-Aldrich) at 25°C for 5 minutes. Tumor cells were mixed with BioPorter–granzyme B complexes for 4 hours at 37°C.Cells were stained for active caspase-3 as an index of apoptosis and examined by flow cytometry.<br>For immune cell tumor infiltration, treated C57BL/6 mice were sacrificed on day 15 following tumor inoculation and tumors were harvested. Tumors were dissected into fragments by cutting, dissociated by a cell strainer. Cell suspensions were stained for intracellular and extracellular protein markers of interest.<br>For analysis of generation of antigen-specific T cells, treated C57BL/6 mice were sacrificed on day 15 following tumor inoculation and tumors, drain lymph nodes and spleens were harvested and incubated with either DMSO or gp100 peptide (KVPRNQDWL) at 1µg/ml for 24hr and stained with anti-CD8. Cell fixation and permeabilization were conducted thereafter with the CytoFix/CytopermTM kit (BD biosciences, San, USA), followed by washing with cold PBS and labeling with anti-IFN-γ. The flow cytometer along with CellQuest Pro software was used to quantify the number of antigen-specific IFN-γ+, CD8+ T cells.
Instrument	FACSVerse flowcytometer (BD Biosciences, Cat no. #651154, year 2014)
Software	Data analysis was performed in BD FACSuite software.
Cell population abundance	N/A
Gating strategy	Tumor cell populations were first gated to exclude cell debris and aggregates based on FSC/SSC. Then cells stained with isotype control were used to determine the boundary between "negative" and "positive" cells; this boundary was used to identify positive cells in samples stained with the specific antibody.

- [x] Tick this box to confirm that a figure exemplifying the gating strategy is provided in the Supplementary Information.
